# Subcellular localization of mutant P23H rhodopsin in an RFP fusion knock-in mouse model of retinitis pigmentosa

**DOI:** 10.1242/dmm.049336

**Published:** 2022-05-06

**Authors:** Michael A. Robichaux, Vy Nguyen, Fung Chan, Lavanya Kailasam, Feng He, John H. Wilson, Theodore G. Wensel

**Affiliations:** 1 Verna and Marrs McLean Department of Biochemistry and Molecular Biology, Baylor College of Medicine, 1 Baylor Plaza, Houston, TX 77030, USA; 2 Departments of Ophthalmology and Biochemistry, West Virginia University, 108 Biomedical Road, Morgantown, WV 26506, USA; 3 Department of Molecular and Human Genetics, Baylor College of Medicine, 1 Baylor Plaza, Houston, TX 77030, USA

**Keywords:** Autosomal dominant, Electron microscopy, Electroretinography, Fluorescence imaging, Retinal degeneration, Rhodopsin

## Abstract

The P23H mutation in rhodopsin (Rho), the rod visual pigment, is the most common allele associated with autosomal-dominant retinitis pigmentosa (adRP). The fate of misfolded mutant Rho in rod photoreceptors has yet to be elucidated. We generated a new mouse model, in which the P23H-Rho mutant allele is fused to the fluorescent protein Tag-RFP-T (P23HhRhoRFP). In heterozygotes, outer segments formed, and wild-type (WT) rhodopsin was properly localized, but mutant P23H-Rho protein was mislocalized in the inner segments. Heterozygotes exhibited slowly progressing retinal degeneration. Mislocalized P23HhRhoRFP was contained in greatly expanded endoplasmic reticulum (ER) membranes. Quantification of mRNA for markers of ER stress and the unfolded protein response revealed little or no increases. mRNA levels for both the mutant human rhodopsin allele and the WT mouse rhodopsin were reduced, but protein levels revealed selective degradation of the mutant protein. These results suggest that the mutant rods undergo an adaptative process that prolongs survival despite unfolded protein accumulation in the ER. The P23H-Rho-RFP mouse may represent a useful tool for the future study of the pathology and treatment of P23H-Rho and adRP.

This article has an associated First Person interview with the first author of the paper.

## Abstract (precis)

**Summary:** A mouse line with a knock-in of the human rhodopsin gene altered to contain the P23H mutation and a red fluorescent protein fusion provides a new model for autosomal-dominant retinitis pigmentosa.

## INTRODUCTION

Retinitis pigmentosa (RP) is a hereditary disease of photoreceptor neurons of the retina that causes night blindness, retinal degeneration and, eventually, complete blindness. RP accounts for half of the known cases of all inherited retinal disease (Daiger et al., 2013), affecting 1:4000 in the USA (Hamel, 2006). Rod and cone photoreceptors, the light-sensing cells in the retina, are polarized neurons with a specialized outer segment (OS) sensory cilium. The OS is the site of phototransduction, the pathway initiated by the photopigment rhodopsin (Rho) in rods and by cone pigments in cones.

Rho is a prototypical G protein-coupled receptor (GPCR) that is densely packaged as an integral transmembrane protein into the lipid bilayers of membrane discs that fill the rod OS cilium. Each mouse OS contains ∼800 discs, and Rho constitutes >90% of the total membrane protein content in the OS (Frederick et al., 2020; Palczewski, 2006). Every day ∼10% of the OS membrane mass in mammalian rods is renewed, as the distal OS discs are shed and engulfed by retina pigment epithelium (RPE) phagocytosis (Bok, 1985; Kevany and Palczewski, 2010), and new discs are generated at the base of the OS (Steinberg et al., 1980; Ding et al., 2015). Thus, in each mouse rod photoreceptor, ∼2.4 million Rho molecules must be synthesized daily in the inner segment (IS), the biosynthetic compartment of rods, to be delivered to the OS cilium to sustain this renewal (Frederick et al., 2020). This trafficking load – including Rho, other visual proteins, and lipids – must pass through a thin, 300 nm connecting cilium (CC) bridge located between the IS and OS.

Correct localization of Rho throughout the highly specialized compartments of rods is necessary to ensure the trafficking rate of Rho molecules and maintain homeostasis and cell survival (Pearring et al., 2013). Thus, rods are susceptible to genetic mutations to Rho itself, which are the leading cause of autosomal-dominant RP (adRP) (Athanasiou et al., 2018). One point mutation that encodes a proline-to-histidine change at codon 23 (P23H) in the N-terminus of Rho is the most common single mutation associated with adRP in North America (Dryja et al., 1990; Sullivan et al., 2006).

P23H-Rho is a misfolding mutation that causes mislocalization of P23H-Rho protein in cell culture from the plasma membrane to dense endoplasmic reticulum (ER) cytoplasmic aggregates (Kaushal and Khorana, 1994; Saliba et al., 2002; Sung et al., 1991). The deleterious effect of the P23H-Rho mutation has been extensively studied in rods in a wide range of animal models, in which it causes rod cell death and retinal degeneration with different degrees of severity (e.g. Lewin et al., 1998; Olsson et al., 1992; Ross et al., 2012). In transgenic P23H-Rho frog rods, the mutant *Xenopus* P23H-Rho protein is retained and mislocalized in the IS ER (Tam and Moritz, 2006), while transgenic bovine P23H-Rho protein in frog rods causes light-induced vesiculation in the IS (Bogea et al., 2015). A zebrafish model expressing mouse P23H-Rho exhibited photoreceptor degeneration, abnormal rod OS formation and P23H-Rho protein mislocalization throughout the malformed rods in adult fish retina (Santhanam et al., 2020).

Transgenic and knock-in P23H-Rho mouse models have variable retinal degeneration rates that coincide with inconsistent photoreceptor cell phenotypes across models (Olsson et al., 1992; Liu et al., 1997; Wu et al., 1998; Sakami et al., 2011; Naash et al., 1993; Frederick et al., 2001; Roof et al., 1994; Kakavand et al., 2020; LaVail et al., 2018). Transgenic P23H-Rho mice generated in a Rho null background have severely dysmorphic rods, in which mutant P23H-Rho is mislocalized in ER surrounding the nucleus (Frederick et al., 2001). In contrast, a knock-in mouse model, featuring a mouse P23H-Rho knock-in allele, had dramatic mutant P23H-Rho protein degradation and no detectable ER mislocalization or accumulation phenotype (Sakami et al., 2011). Rod degeneration in the P23H-Rho knock-in heterozygotes was fairly rapid [50% rod loss before P40 (Sakami et al., 2014)] but even more severe in homozygotes. The undegraded P23H-Rho protein in the P23H-Rho knock-in rods normally localized to the OS and caused abnormal OS disc formation (Sakami et al., 2011, 2014). One challenge for characterizing the fate of misfolded P23H-Rho and testing its effects on different therapeutic approaches, is the difficulty in distinguishing wild-type (WT) rhodopsin from a variant that differs by one amino acid residue. We previously generated a P23H-Rho-GFP fusion knock-in mouse to easily visualize the mutant protein in rods, and we observed a gross mislocalization of mutant P23H-Rho-GFP in the IS (Price et al., 2011). Furthermore, we found that the mutant fusion protein was largely degraded; however, we did not characterize any subcellular phenotypes in single rods on a nanoscopic level in the P23H-Rho-GFP mouse.

We have generated a new P23H-Rho mouse model in order to study mutant P23H-Rho protein localization and dynamics, and to serve as a platform for testing gene-based and other therapies. The latter goal is best achieved with a model that has a relatively slow rate of retinal degeneration in line with the slowly progressive course of human vision loss in adRP, to permit long-term studies. Also, a relatively non-perturbing tag facilitates the testing of therapeutic interventions and strategies.

In this new knock-in mouse model, the mutant P23H-Rho protein is fused to a photostable Tag-RFP-T (Shaner et al., 2008) fusion tag, which we have used to study the mislocalization of the protein. We have also studied the morphological and functional changes that accompany a slowly progressing retinal degeneration.

## RESULTS

### Generation of the P23H-hRho-RFP knock-in mouse

We introduced into the mouse *Rho* locus, the human *RHO* (indicated as ‘hRho’) gene (introns and exons) encoding both the P23H mutation in the first exon and a fusion to Tag-RFP-T at the C-terminus. Tag-RFP-T is a red fluorescent protein (excitation maximum, 555 nm; emission maximum, 584 nm) based on a naturally occurring anemone protein engineered for both bright fluorescence and enhanced photostability (Shaner et al., 2008). Notably, Tag-RFP-T fluorescence is compatible with GFP fluorescence for multiplex detection, and it has been used for multiple applications since its design, including as a fluorescent fusion marker (Grassart et al., 2014; Boucrot et al., 2012; Weber et al., 2020).

**Fig. 1.**
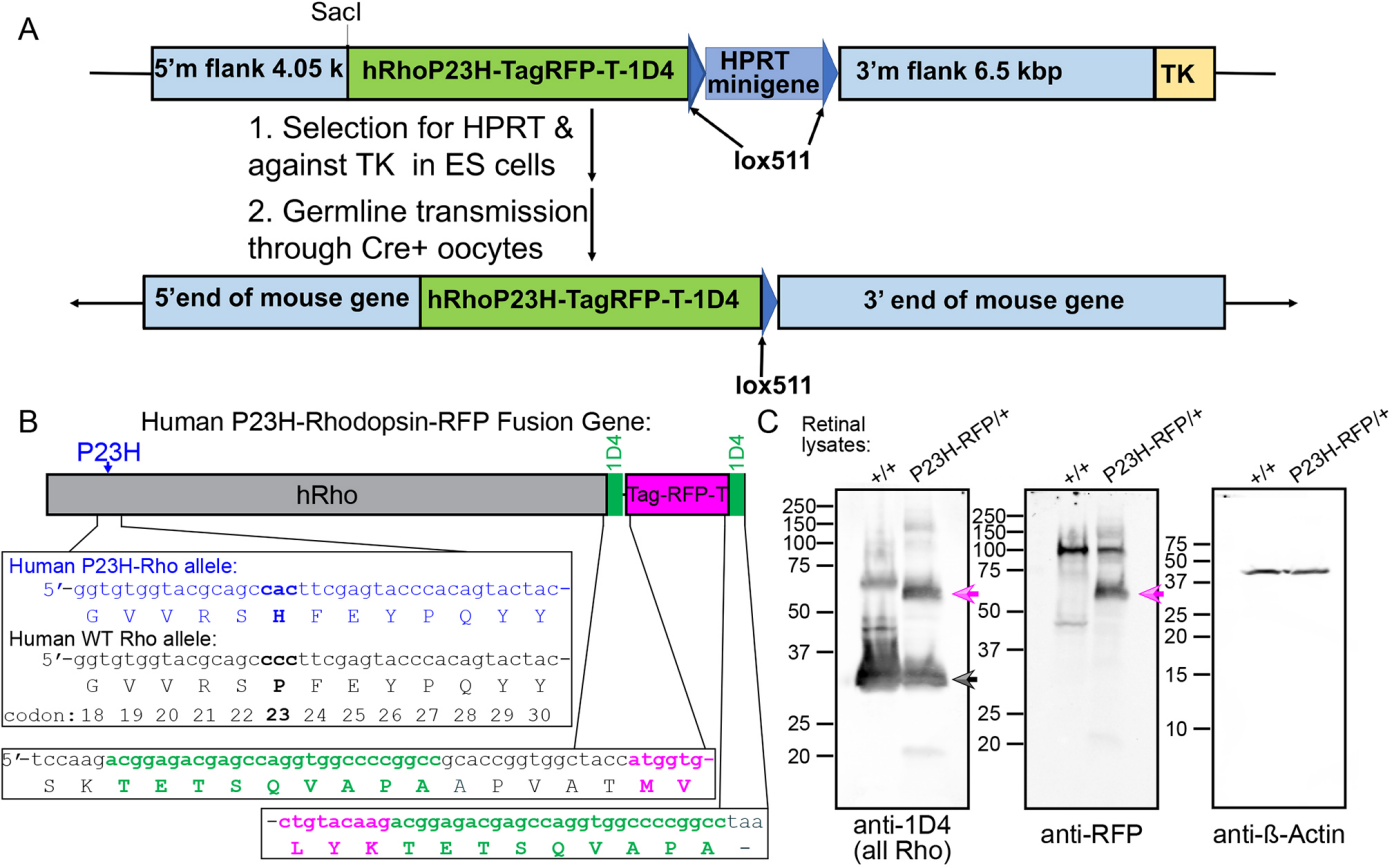
**Construction and validation of the P23H-hRho-TagRFP-T knock-in mouse.** (A) Targeting construct used in embryonic stem (ES) cells and resulting gene structure. (B) Map of knock-in human P23H-rhodopsin-RFP fusion gene. A portion of the sequence of exon 1 from the P23H-Rho allele in the fusion gene (blue) is aligned to the same human wild-type (WT) *RHO* (indicated as ‘hRho’) allele sequence (black). The mutated codon 23 is in bold font. The transition sequence from the 1D4 terminal signal sequence to the Tag-RFP-T with a short linker sequence is shown in the middle panel, and the C-terminal sequence with extra 1D4 epitope sequence is appended to the end of Tag-RFP-T before the stop codon. (C) Immunoblot confirmation of the P23HhRhoRFP fusion protein expression in P23H-RFP/+ heterozygous retinas. Retinal lysates from WT (+/+) mouse, at P22, and P23H-RFP/+ mouse, at P45; 100 µg of total protein from each lysate was loaded onto SDS-PAGE gels. Blot membranes were probed with either of the following antibodies: anti-1D4, anti-RFP or anti-β-actin (a loading control). Blot scans for the protein ladder were used to mark molecular mass (MW). The ∼65 kDa P23HhRhoRFP fusion protein band is present in the P23H-RFP/+ lane in both anti-1D4 and anti-RFP blot scans (magenta arrows). Monomeric mouse Rho protein band is in both lanes in the anti-1D4 blot scan (black arrow). Higher MW species are formed by rhodopsin multimerization.

We also added an additional 1D4 signaling sequence to the carboxy-terminus of the P23H-hRho+Tag-RFP-T fusion allele (Fig. 1A,B). Our rationale was that the C-terminal RFP fusion tag in the expressed P23H-hRho-Tag-RFP-T (hereafter P23HhRhoRFP) protein may inhibit the endogenous Rho 1D4 sequence, leading to artifacts not attributable to the P23H mutation. The additional 1D4 sequence was also shown to be necessary for normal trafficking of transgenic Rho-Dendra fusion protein in *Xenopus laevis* rods (Lodowski et al., 2013).

**Fig. 2.**
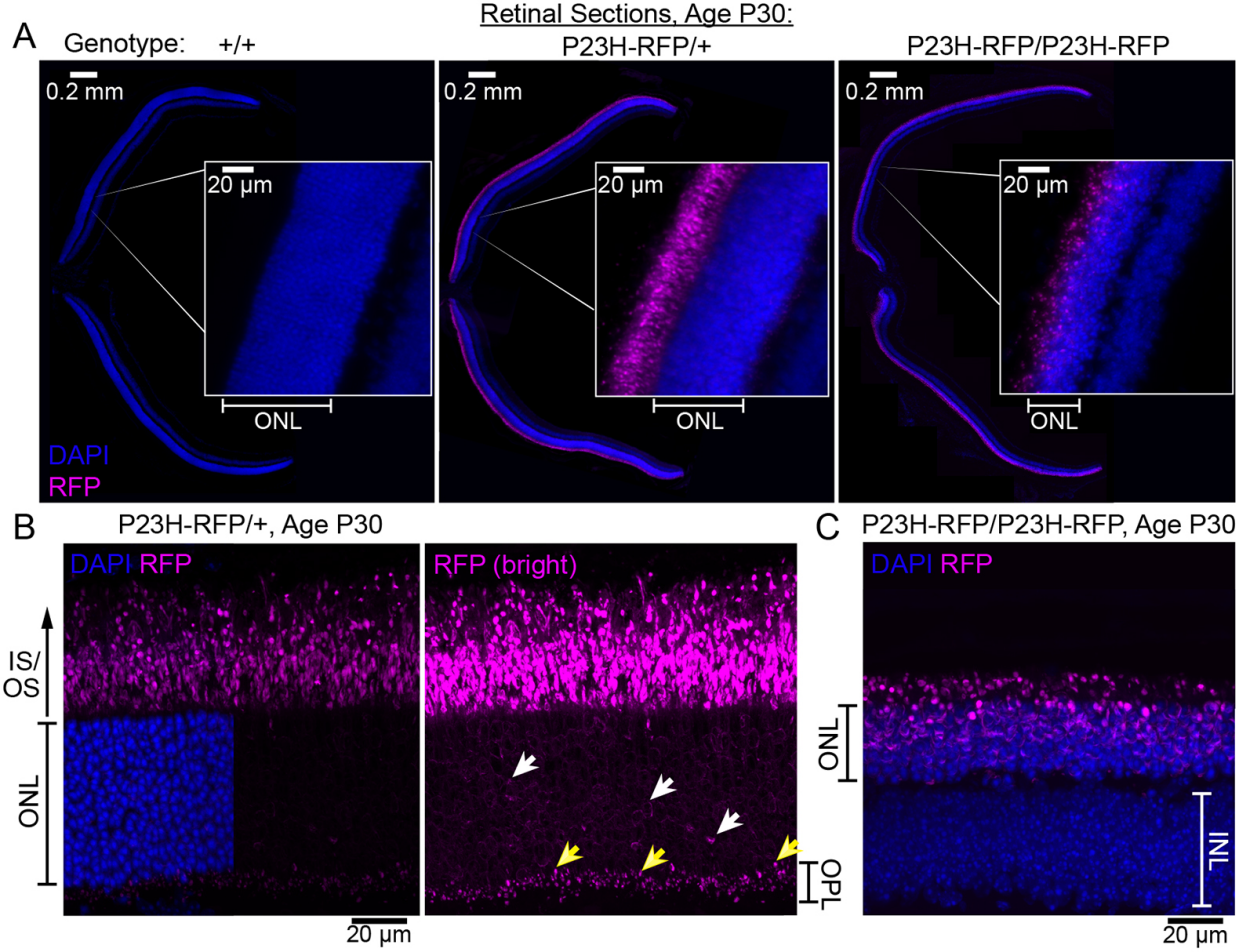
**Localization of RFP fluorescence in the P23H-hRho-RFP knock-in mouse retina.** (A) Widefield fluorescence images of mouse retinal cryosections from age-matched P30 WT (+/+), heterozygous (P23H-RFP/+) and homozygous knock-in mice (P23H-RFP/P23H-RFP). Sections were counterstained with DAPI to label nuclei in the retina (blue). RFP fluorescence in the outer nuclear layer (ONL) of each retina, which is the location of the DAPI+ photoreceptor nuclei, is demarcated. Insets are magnified views of the indicated regions. (B) Confocal *z*-projection of a retinal cryosection from a P30 heterozygous P23H-RFP/+ mouse. The left panel is gain adjusted to avoid pixel saturation and split into two. On the left, the DAPI and RFP channels are overlaid, and on the on the right, only the RFP signal is shown. The brightest RFP signal is in the outer photoreceptor layers, the inner segment (IS) and outer segment (OS) region, where P23HhRhoRFP protein is localized in aggregates. In the same image, with the gain of the RFP signal raised to saturation (right panel), additional P23HhRhoRFP protein is observed in the rod photoreceptor synapses within the outer plexiform layer (yellow arrows). To a lesser degree, P23HhRhoRFP is also localized in the ONL, in the cytoplasm surrounding the photoreceptor nuclei (white arrows). (C) Confocal *z*-projection through a retinal cryosection from a P30 P23H-RFP/P23H-RFP homozygote. The width of the DAPI-stained ONL is thinner compared to that in the heterozygote. In the homozygous retina, mutant P23HhRhoRFP fusion protein is also more prominently localized in the ONL compared to in the heterozygote. INL, inner nuclear layer.

The P23H-hRho-TagRFP knock-in mice were generated the same way we previously generated P23H-hRho-GFP knock-in mice (Price et al., 2011), by gene targeting in the *Hprt*^−/−^ embryonic stem (ES) cell line AB2.2 123, which was derived from mouse strain 129SvEv, essentially as described previously (Chan et al., 2004, 2011). We introduced the P23H mutation into the targeting vector by site-directed mutagenesis (QuikChange^®^, Stratagene). An ISceI recognition site was engineered into the middle of the first intron in the rhodopsin gene at position 1340 from the start of translation, but it was not used in the experiments described here. The Darwin Transgenic Core Facility, Baylor College of Medicine, electroporated ES cells, selected for HPRT^+^TK^−^ cells and injected correctly targeted ES cells into blastocysts from albino C57BL/6-Tyr^c-Brd^ mice (Zheng et al., 2000). Founder mice carrying the HPRT-P23H-hRho-TagRFP allele were crossed to GDF-9-iCre mice (Lan et al., 2004) to remove the *HPRT1* minigene. P23H-hRho-TagRFP (hereafter ‘P23H-RFP’ in reference to the knock-in allele) mice were extensively backcrossed to C57BL/6 mice. We confirmed that the knock-in was successful by sequencing genomic DNA from the knock-in mouse. We verified expression of the P23HhRhoRFP fusion by fluorescence microscopy of retinas and by immunoblotting (Figs 1 and 2).

We used P23H-RFP/+ heterozygous retinas to examine the expression of the mutant fusion protein alongside the WT mouse Rho protein from the WT allele (Fig. 1C). Both WT mouse Rho protein and the product of the knock-in allele were detected in P23H-RFP/+ retinal lysates by probing with the anti-1D4 monoclonal antibody. The P23HhRhoRFP fusion protein was detected as a strong ∼65 kDa band in P23H-RFP/+ lysates using both anti-1D4 and anti-RFP antibodies, verifying robust expression.

### P23HhRhoRFP protein was mislocalized in rod photoreceptor neurons

We next tested the fluorescence pattern of the P23HhRhoRFP fusion protein in the retinas of both P23H-RFP/+ heterozygous and P23H-RFP/P23H-RFP homozygous mice. RFP fluorescence was evident in the outer photoreceptor layers of retinal sections from both heterozygotes and homozygotes at postnatal day (P)30 (Fig. 2A). In a confocal *z*-projection of a retinal section from a P30 P23H-RFP/+ heterozygote, P23HhRhoRFP was most prominently located in brightly fluorescent puncta or ‘aggregates’ within the regions of the ISs and OSs of photoreceptors (Fig. 2B). Less prominent but visible in this same section was P23H-Rho-RFP fluorescence at the photoreceptor synapses of the outer plexiform layer (OPL) and in the cytoplasm surrounding the photoreceptor nuclei of the outer nuclear layer (ONL) (Fig. 2B). Compared to both WT and heterozygotes, the ONL of P30 homozygotes was noticeably thinner based on 4′,6-diamidino-2-phenylindole (DAPI)^+^ nuclei staining (Fig. 2C). P23HhRhoRFP aggregates were also clearly visible in the ONL of the homozygous retina.

We crossed our new P23H-RFP mouse line with WT hRho-GFP mouse lines for dual fluorescent tag multiplex imaging. This approach allowed us to investigate the subcellular dynamics of the mutant P23HhRhoRFP protein relative to hRho-GFP without the P23H mutation. In addition to the hRho-GFP fusion mouse that we previously reported (Chan et al., 2004), we also generated a new line with an additional 1D4 signal sequence added to the end of the EGFP sequence (‘hRho-GFP-1D4’). As heterozygotes, we could not discriminate any phenotypic difference between these GFP fusion mice.

**Fig. 3.**
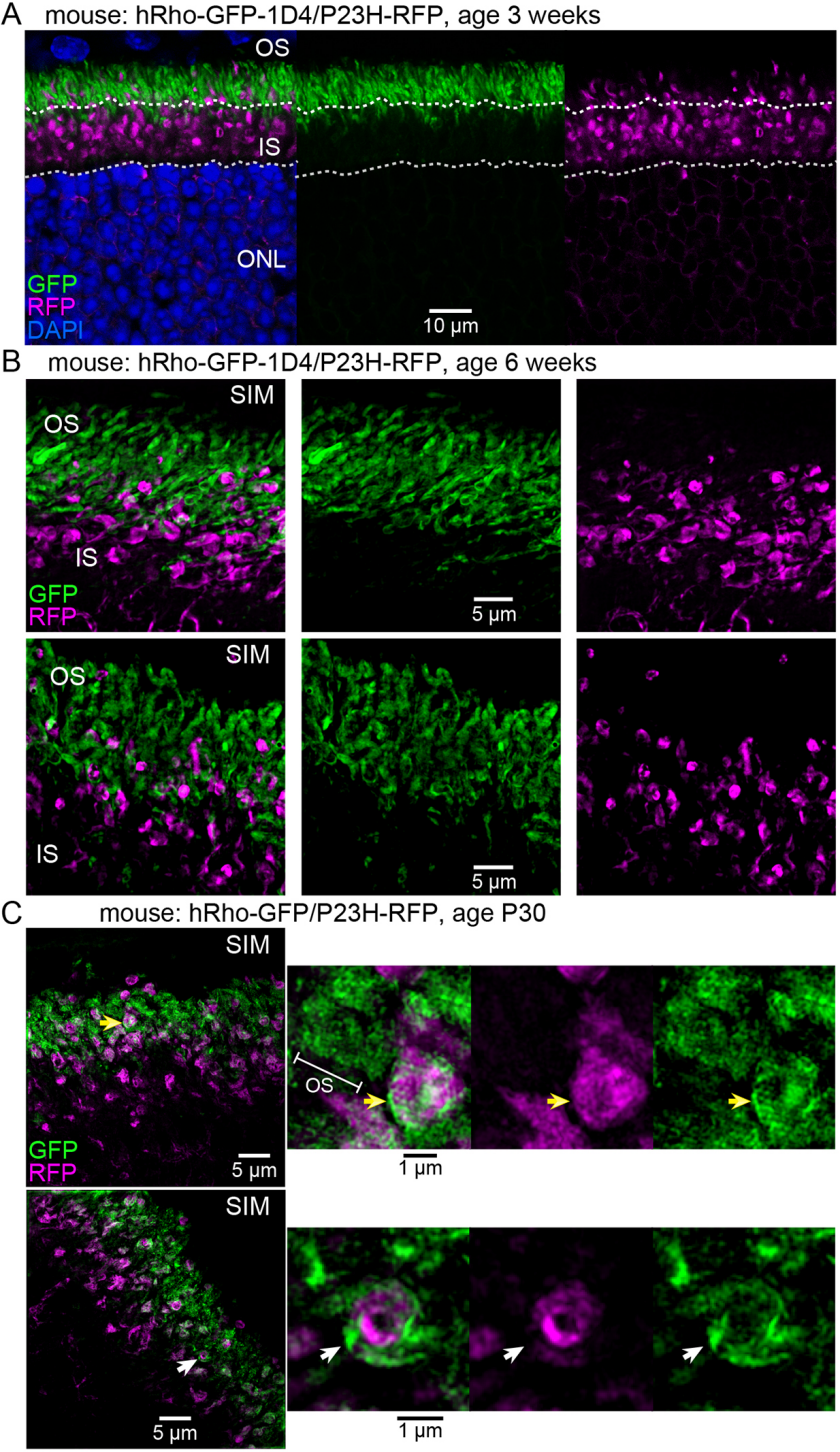
**P23HhRhoRFP^+^ fusion aggregates are localized in the ISs of rod photoreceptor neurons.** (A) Confocal *z*-projection of a section from a hRho-GFP-1D4/P23H-RFP heterozygous mouse retina, at 3 weeks of age. WT hRho-GFP fusion (green), which has an additional C-terminal 1D4 signal sequence, is correctly localized to the OSs of rods. RFP^+^ aggregates containing mutant P23HhRhoRFP protein (magenta) are primarily located in the ISs of rods and almost entirely segregated from the GFP^+^ OS layer. White dotted lines demarcate the OS/IS boundary (upper) and IS/ONL boundary (lower). (B) Structured illumination microscopy (SIM) micrographs of retinal sections from the same line as A at 6 weeks. GFP^+^ OS and RFP^+^ IS aggregates are segregated with no apparent colocalization. (C) In SIM images of retinal sections from an alternate GFP/RFP heterozygote at P30, in which the WT hRho-GFP fusion does not have an additional 1D4 signal peptide, the GFP fluorescence is not exclusively located in the OS layer, but rather is partially mislocalized with P23H-hRho-RFP. In a magnified example, hRho-GFP is colocalized around and within a P23HhRhoRFP aggregate (yellow arrows). In another magnified example, hRho-GFP is wrapped around an P23HhRhoRFP aggregate (white arrows).

Both GFP fusion lines were crossed to our new P23H-RFP knock-in mouse. In confocal images of retinal sections from an adult hRho-GFP-1D4/P23H-RFP heterozygote, we observed a drastic localization difference between hRho-GFP-1D4, which correctly populated the rod photoreceptor OS cilia, and the P23HhRhoRFP aggregates, which were prominently mislocalized in the rod IS layer (Fig. 3A). We examined retinal sections from this same hRho-GFP-1D4/P23H-RFP heterozygous mouse line with structured illumination microscopy (SIM) super-resolution imaging and observed a clear segregation of P23HhRhoRFP from the hRho-GFP-1D4 in the OS cilia (Fig. 3B). Interestingly, in retinal sections from the other heterozygous hRho-GFP/P23H-RFP mice, we found evidence of hRho-GFP mislocalization in the same region as the P23HhRhoRFP aggregates, in what appear to be distinct but interwoven membrane compartments (Fig. 3C). This result suggests that the added C-terminal 1D4 sequence prevents hRho-GFP from being mislocalized with P23HhRhoRFP in the GFP/RFP heterozygous animals.

**Fig. 4.**
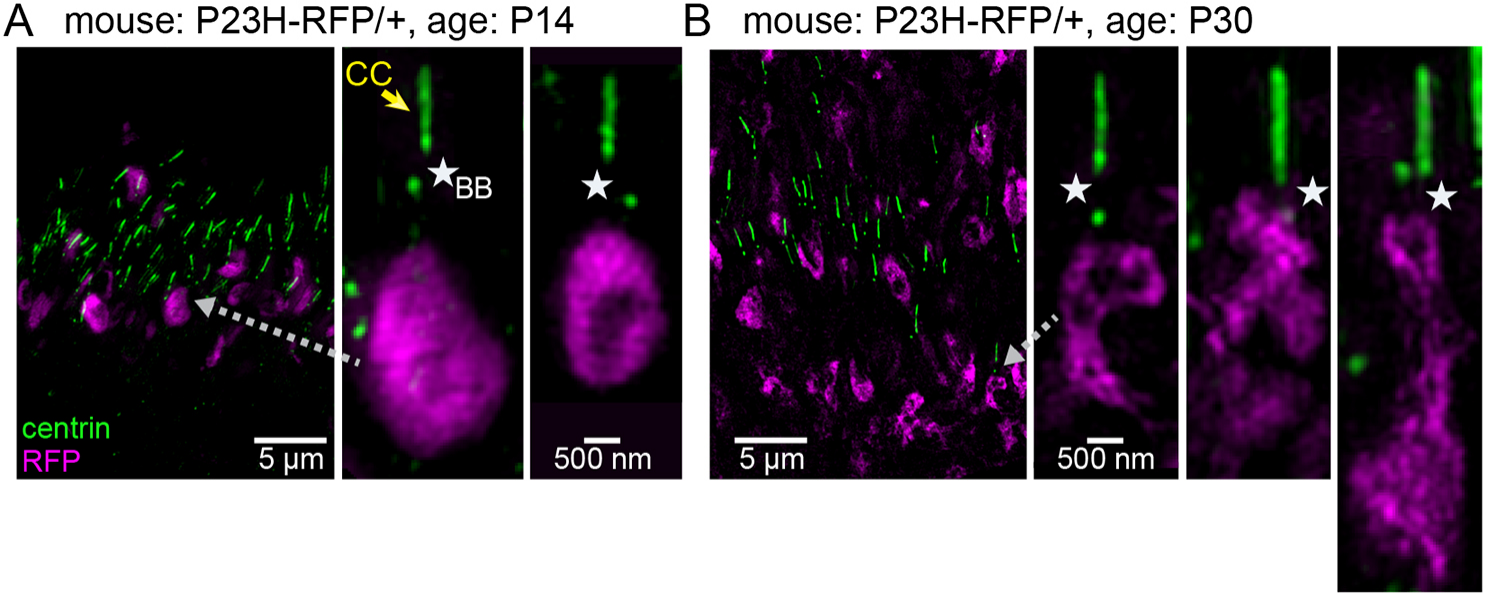
**Mutant P23H-hRho-RFP IS aggregates are localized near the basal body (BB) in rod neurons.** (A) In a SIM *z*-projection image of a retinal section from the P23H-RFP/+ mouse at P14, centrin immunolabeling (green) marks the location of rod connecting cilium (CC) and BB relative to the P23HhRhoRFP fluorescent aggregates (magenta). In magnified views, single RFP^+^ IS aggregates are located just proximal to the CC (yellow arrow) and BB (white stars). The BB region is demarcated by the centrin^+^ mother centriole at the proximal end of the CC and the daughter centriole, which is a separated centrin^+^ punctum beneath the mother centriole. (B) SIM image of a retinal section from the P23H-RFP/+ mouse at P30 also with centrin immunolabeling. In magnified views, the RFP^+^ aggregates at P30 are still generally located proximal to the BB (white stars), with some examples of RFP overlapping with the BB region. The organization of the RFP^+^ aggregates in these P30 rods is less compact and more reticulated compared to that of RFP^+^ aggregates in P14 rods (see examples in A). Gray dotted line arrows mark a region that is magnified from the same image.

To visualize the location of the P23HhRhoRFP IS aggregates relative to the CC and basal body (BB), we used centrin as an antibody marker for the CC and BB (Trojan et al., 2008; Robichaux et al., 2019) in retinal sections from P23H-RFP/+ mice and imaged by SIM. At P14, we found many examples of P23HhRhoRFP aggregates that were located just proximal to the BB (Fig. 4A). We observed this same sub-BB localization of the RFP aggregates in retinal sections from P30 P23H-RFP/+ mice (Fig. 4B), with some examples of P23HhRhoRFP fluorescence overlapping into the BB region. In retinas from both ages, the RFP fluorescence pattern within these P23HhRhoRFP clumps was discontinuous with dark patches. Morphologically, the P23HhRhoRFP fluorescent aggregates in P14 retina had a compact and defined elliptical shape compared to the aggregates in the P30 retina, which appeared more elongated and less structured.

Taken together, fluorescence images of P23H-RFP retinas at different ages demonstrate that the P23HhRhoRFP mutant fusion protein was excluded from the OS and mislocalized within aggregates near the BB in the IS and to a lesser degree in the ONL and the photoreceptor synapses. This result suggests that the mutant fusion protein is accumulating in a trafficking stage just prior to the BB and integration into the cilia, and thus it fails to be properly transported to the OS.

### P23H-RFP/+ mice have mild and gradual retinal degeneration

**Fig. 5.**
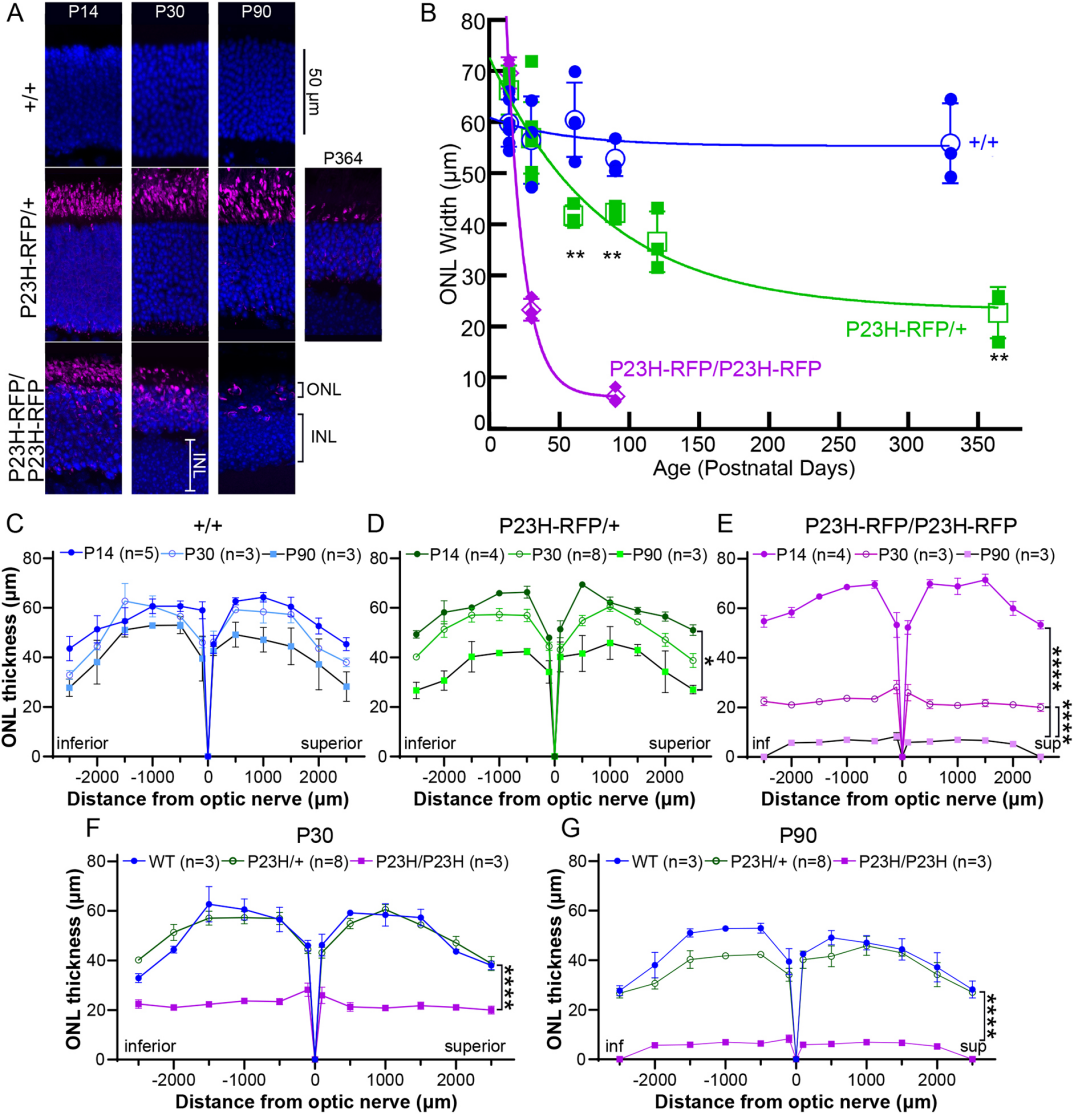
**Time course of retinal degeneration in P23H-RFP/+ heterozygous and P23H-RFP/+ homozygous mice.** (A) Confocal *z*-projection images of retinal cryosections from WT (+/+), P23H-RFP heterozygote (het), and P23H-RFP/P23H-RFP homozygous (homo) mice at the indicated times. DAPI (blue) labels photoreceptor nuclei in the ONL and the bipolar cell nuclei in the INL. RFP fluorescence (magenta) is primarily located in the IS layers of het retinas at all time points, while RFP is also located in the ONL in homo retinas. At P90, the homo ONL is reduced to very few disorganized nuclei surrounded by RFP. (B) Time course plot of ONL thickness between genotypes. Measurements correspond to the ONL thickness of the retina 500 µm inferior to the optic nerve. Unfilled shapes correspond to the mean value, and error bars signify s.e.m. Solid lines represent fits to exponential decays to plateau, as described in the Materials and Methods. Unpaired two-tailed Student's *t*-tests were calculated to compare +/+ and het values for significance. Comparisons with significant differences are as follows: P61 +/+ versus P60 het (**P=0.0081), P90 +/+ versus P90 het (**P=0.0072), and P330 +/+ versus P364 het (**P=0.0035). (C) ‘Spider’ plots of ONL thickness for 13 positions in +/+ retinal cryosections from the P14, P30 and P90 time points, spanning positions from the optic nerve to the inferior and superior retina. Positions directly adjacent to the optic nerve position (‘0’) correspond to 100 µm superior and inferior to the optic nerve. The most peripheral positions of each plot correspond to 100 µm from the superior and inferior ends of the retina. For each position on the plots, circles correspond to mean values and error bars signify s.e.m. (D) Spider plots for P14, P30 and P90 P23H-RFP/+ het retinas. Statistical tests of differences between plots from different time points were performed using two-way ANOVA with Šídák multiple comparisons test. The only comparison with significance is P14 het versus P90 het (*P=0.0215). (E) Spider plots for P23H-RFP homozygous retinal cryosections at the same time points. The same statistical tests as in D revealed significant differences for the following comparisons: P14 versus P30, P14 versus 90, and P30 versus P90 (all ****P<0.0001). (F) Spider plots for all retinal cryosections at P30 to compare differences between genotypes. The same tests as in D and E revealed significant differences at P30 for +/+ versus homo and het versus homo (both ****P<0.0001). (G) Spider plots to compare genotypes at P90. By the same tests as above, there were significant differences at P90 for +/+ versus homo and het versus homo (both ****P<0.0001).

Next, we tested the effect of the mutant P23HhRhoRFP fusion on retinal health by measuring the rates of retinal degeneration in both P23H-RFP/+ heterozygous and P23H-RFP/P23H-RFP homozygous mice. We measured the thickness of the ONL, in which the photoreceptor nuclei were stained for DAPI fluorescence, in retinal sections from both genotypes and in WT (+/+) control retinas from mice at multiple time points. Overall, the P23H-RFP/+ heterozygous retinas degenerated more slowly over time compared to the homozygous retinas, as measured by ONL thickness (Fig. 5). Our analysis covered the full retina – peripheral to central – to test for any region-specific degeneration caused by the mutant P23HhRhoRFP fusion protein.

At P14, there was no difference in the ONL thickness in the retinas among any of the phenotypes, indicating that the retinas develop normally in the P23H-RFP heterozygous and homozygous mutants (Fig. 5A-E). Thereafter, the width of the ONL in the heterozygous retinas declined slowly, approaching a final value of 23 μm with a time constant of 56 days, whereas the ONL in the homozygous mutants declined much more rapidly, with a time constant of 12 days. By P90, the ONL in P23H-RFP/P23H-RFP homozygous retinas was reduced to a single, disorganized layer of nuclei (Fig. 5A,E,G).

Although the ONL thickness in P23H-RFP/+ heterozygous retinas was significantly reduced in the inferior retina at P60 and P90 (Fig. 5B,G), the thickness across all regions of the retina in P23H-RFP/+ mutants was not significantly different from that of +/+ retinas at P90 (Fig. 5G). Notably, compared to P14 P23H-RFP/+ measurements, the P90 P23H-RFP/+ ONL thickness was significantly reduced (Fig. 5D). By P364, it was evident that the ONL had been severely reduced in P23H-RFP heterozygous retinas due to nuclei loss (Fig. 5B, see example in Fig. 5A); the ONL width at 364 days in the heterozygotes was 40% of that in WT at 360 days.

### Electroretinogram (ERG) rod photoreceptor function was moderately diminished in P23H-RFP/+ mice

**Fig. 6.**
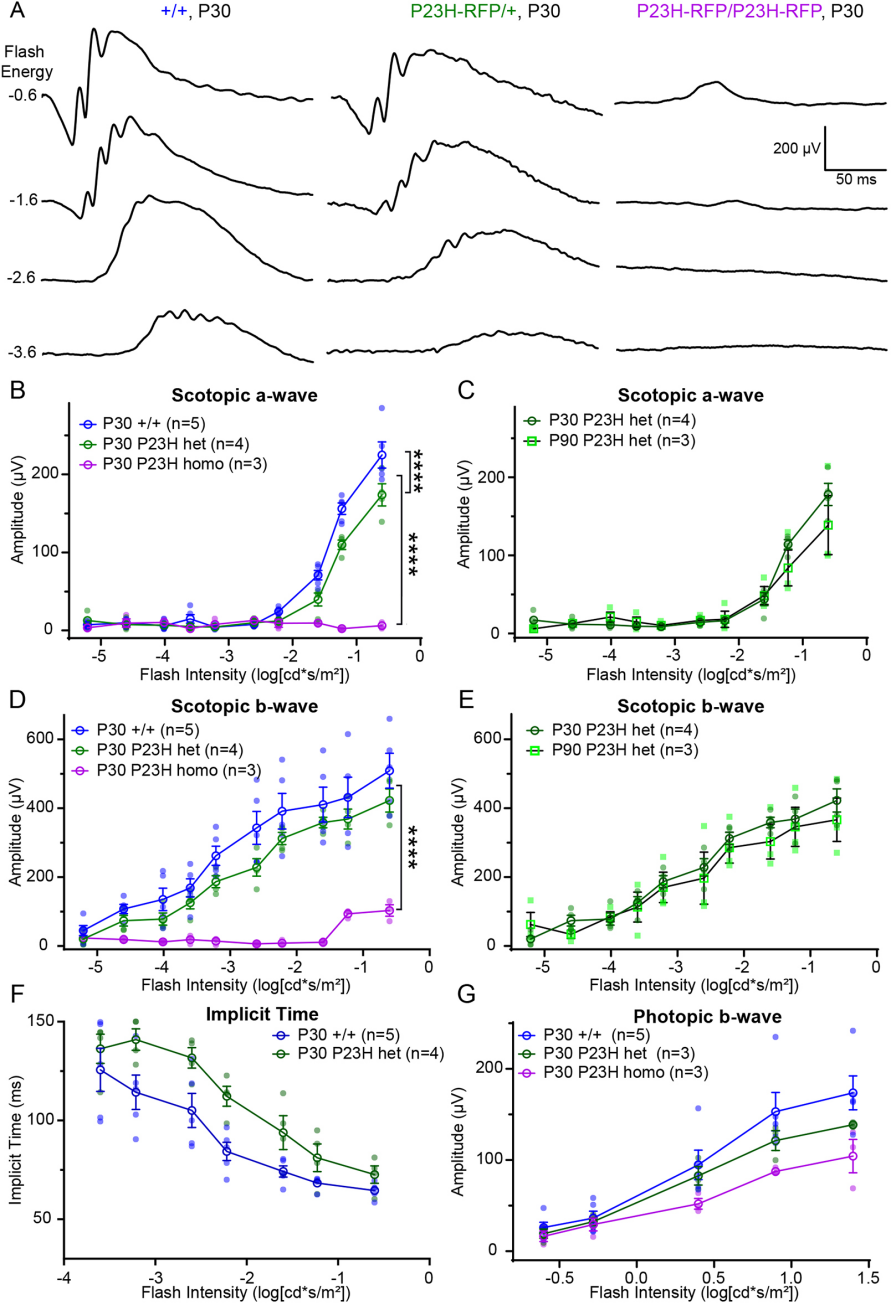
**P23H-RFP/+ heterozygous mice have slightly reduced rod photoreceptor electroretinogram (ERG) responses.** (A) Example ERG recordings from +/+ WT, P23H-RFP/+ het and P23H-RFP/P23H-RFP homo mice at P30. (B) Aggregate of P30 scotopic a-wave amplitudes. In all plots, solid shapes are data points, empty shapes signify mean values, and error bars signify s.e.m. Statistical comparison tests of all the ERG data were performed using two-way ANOVA with Šídák multiple comparisons tests. All statistical comparisons of P30 a-wave amplitudes showed significance: +/+ versus het (****P<0.0001), +/+ versus homo (****P<0.0001), and het versus homo (****P<0.0001). (C) Scotopic a-wave amplitudes between P30 and P90 P23H/+ het mice were not statistically different (*P*=0.27). (D) Aggregate of P30 scotopic b-wave amplitudes. Statistically significant differences were calculated for +/+ versus homo (****P<0.0001) and het versus homo (****P<0.0001). WT versus het was not statistically different (*P*=0.9744). (E) Scotopic b-wave amplitudes between P30 and P90 P23H/+ het mice were not statistically different (*P*=0.9375). (F) Aggregate implicit times, the time to peak scotopic b-wave post a-wave, in P30 +/+ and het mice. P30 het mice had higher implicit times than P30 +/+ mice at intermediate flash stages, but the difference over the entire flash range was not statistically significant (*P*=0.5651). (G) Aggregate of P30 photopic b-wave amplitudes. There were no statistically significant differences among the genotypes: +/+ versus het (*P*=0.749), +/+ versus homo (*P*=0.1298), het versus homo (*P*=0.1553).

We next used ERG recordings to test and correlate retina visual function to the mild and severe retinal degeneration phenotypes in P23H-RFP/+ mice and P23H-RFP/P23H-RFP mice, respectively. We found generally that ERG waveforms at P30 correlated with the retinal degeneration phenotypes of the mutant P23H-RFP genotypes compared to +/+ control mice (Fig. 6A). In dark-adapted conditions, the scotopic a-wave amplitudes in P23H-RFP/+ mice were significantly reduced compared to those in +/+ mice at P30 (Fig. 6B); however, the a-waves stabilized over time and were not further diminished in P23H-RFP/+ mutants at P90 compared to those at P30 (Fig. 6C). Scotopic b-wave values were not significantly reduced in P30 P23H-RFP/+ compared to +/+ mice (Fig. 6D), and, like the a-wave, the b-wave values were not diminished in P23H/+ mice at P90 compared to those at P30 (Fig. 6E). We also measured the implicit times from scotopic ERG recordings, which are the times from the a-wave deflection to peak b-wave. In P30 P23H-RFP/+ mice compared to P30 +/+ mice, implicit times were higher at moderate flash intensities, but there was no significant difference over the entire flash range (Fig. 6F).

P23H-RFP/P23H-RFP homozygous mice have essentially no scotopic ERG response (Fig. 6A,B,D). The minor b-wave response at high flash intensities in homozygotes could be attributed to cones. We also measured the cone ERG response in light-adapted mice from all genotypes at P30. Photopic b-wave amplitudes were recorded in both P23H-RFP/+ and P23H-RFP/P23H-RFP mice, and although they appeared slightly lower in amplitude, they were not significantly reduced compared to those in +/+ mice (Fig. 6G).

**Fig. 7.**
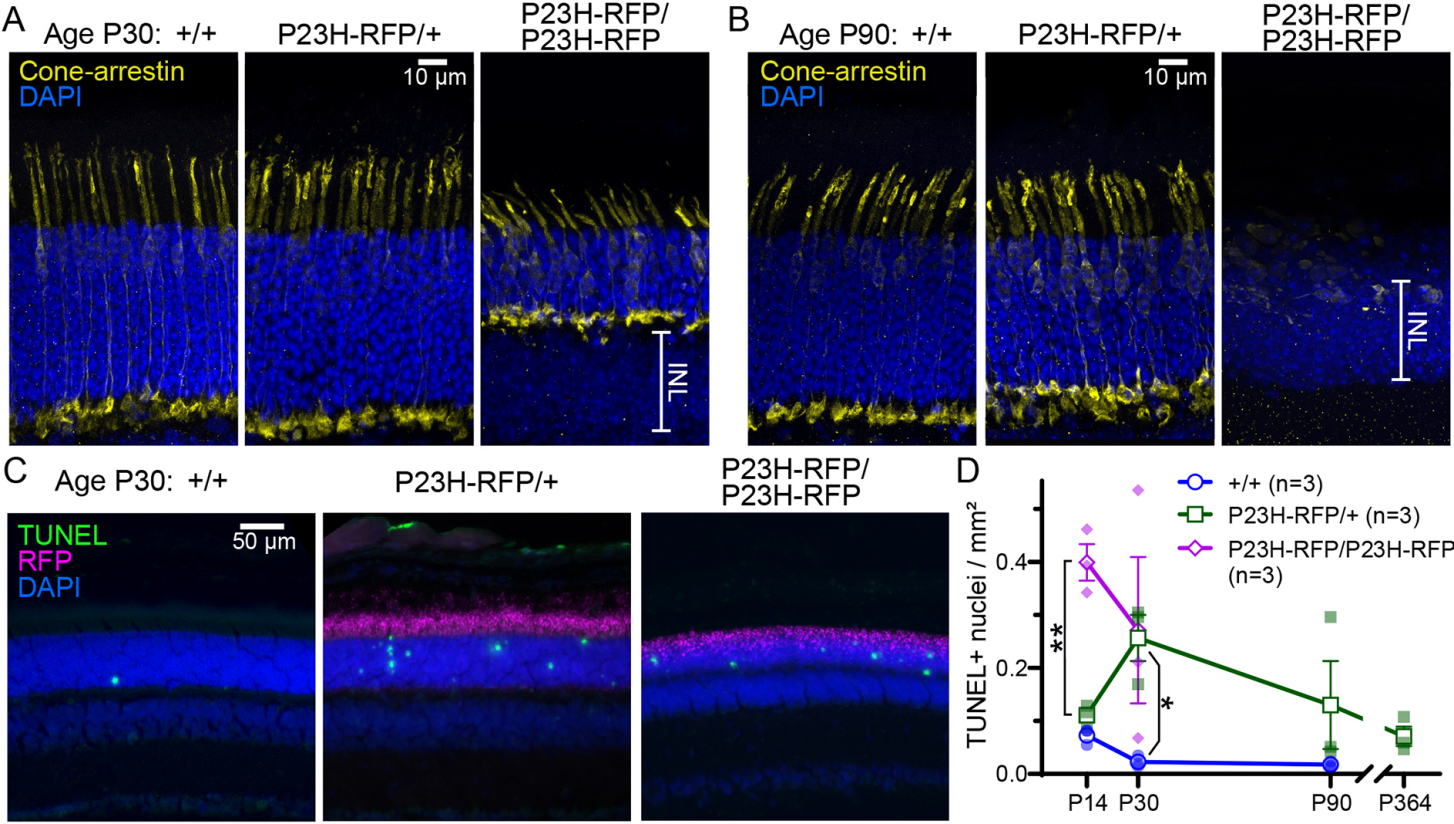
**Cone immunolocalization and TUNEL analysis of photoreceptor cell death in P23H-hRho-RFP retinas.** (A,B) Examples of cone arrestin immunofluorescence staining (yellow) in retinal sections at P30 (A) and P90 (B), among +/+, P23H-RFP/+ and P23H-RFP/P23H-RFP mice. DAPI staining (blue) labels both photoreceptor nuclei in the ONL and in the INL. (C) TUNEL fluorescence analysis of photoreceptor cell in retinal cryosections. Shown are examples of P30 retinal sections from +/+, P23H-RFP/+ het and P23H-RFP/P23H-RFP homo mice with TUNEL^+^ nuclei (green) within the DAPI-stained nuclei of the ONL (blue). RFP fluorescence is magenta. (D) Time course plot of aggregate TUNEL^+^ nuclei/mm^2^ measurements among all genotypes at multiple time points. Statistical comparisons among groups were performed using unpaired two-tailed Student's *t*-tests. At P14, homo retinas have statistically more TUNEL^+^ nuclei compared to both +/+ (*P=0.0167) and het (**P=0.0098) retinas. At P30, het retinas have statistically more TUNEL^+^ nuclei compared to +/+ retinas (*P=0.042), but the rate of TUNEL^+^ nuclei between het and +/+ retinas is not statistically different at P90 (*P*=0.312).

To determine whether there were any visible alterations of cone morphology in the mutants, we used cone arrestin immunofluorescence labeling in our P23H-RFP mice at P30. Cones populated the retinas of P23H-RFP/+ heterozygotes as well as in the retinas of P23H-RFP/P23H-RFP homozygotes despite the massive loss of photoreceptor nuclei in the homozygotes (Fig. 7A). At P90, we still observed cones in P23H-RFP/+ heterozygotes, but in P90 homozygotes there were no visible cones remaining, presumably as a result of the nearly complete loss of rods (Fig. 7B).

To determine the rate of cell death in our P23H-RFP mice, we used terminal deoxynucleotidyl transferase dUTP nick end labeling (TUNEL) fluorescence. We observed TUNEL^+^ photoreceptor nuclei in the ONL of retinal sections from both P23H-RFP/+ and P23H-RFP/P23H-RFP mice (Fig. 7C). We measured the number of TUNEL^+^ nuclei per mm^2^ area of the ONL at multiple time points. At P14, the number of TUNEL^+^ nuclei in P23H-RFP/+ heterozygous retinal sections was comparable to that in +/+ retinal sections, while the rate was significantly greater in homozygous retinal sections at P14 (Fig. 7D). By P30, the number of TUNEL^+^ nuclei was significantly greater in P23H-RFP/+ retina compared to +/+ retina (Fig. 7D); however, at P90, the TUNEL^+^ density in heterozygotes was no longer significantly different from that in +/+ mice (Fig. 7D).

Overall, we observed that P23H-RFP/+ heterozygous mice have a slow and partial retinal degeneration and mild loss of rod ERG function. At P90, the heterozygous retinas were still comparable in overall health to control +/+ retinas, despite the gross misaccumulation of P23HhRhoRFP protein in the rod ISs. The rate of photoreceptor cell death in P23H-RFP/+ retinas indicates that a moderate burst of degeneration begins after P14, which has slowed by P90. By comparison, the retinal degeneration and ERG phenotypes in homozygous P23H-RFP/P23H-RFP mice were much more severe, such that, at P90, nearly all photoreceptor neurons were lost in the retinas of homozygotes.

### Quantification of select mRNA levels from P23H-RFP/+ mouse retinas by quantitative reverse transcription PCR (Q-RT-PCR)

**Fig. 8.**
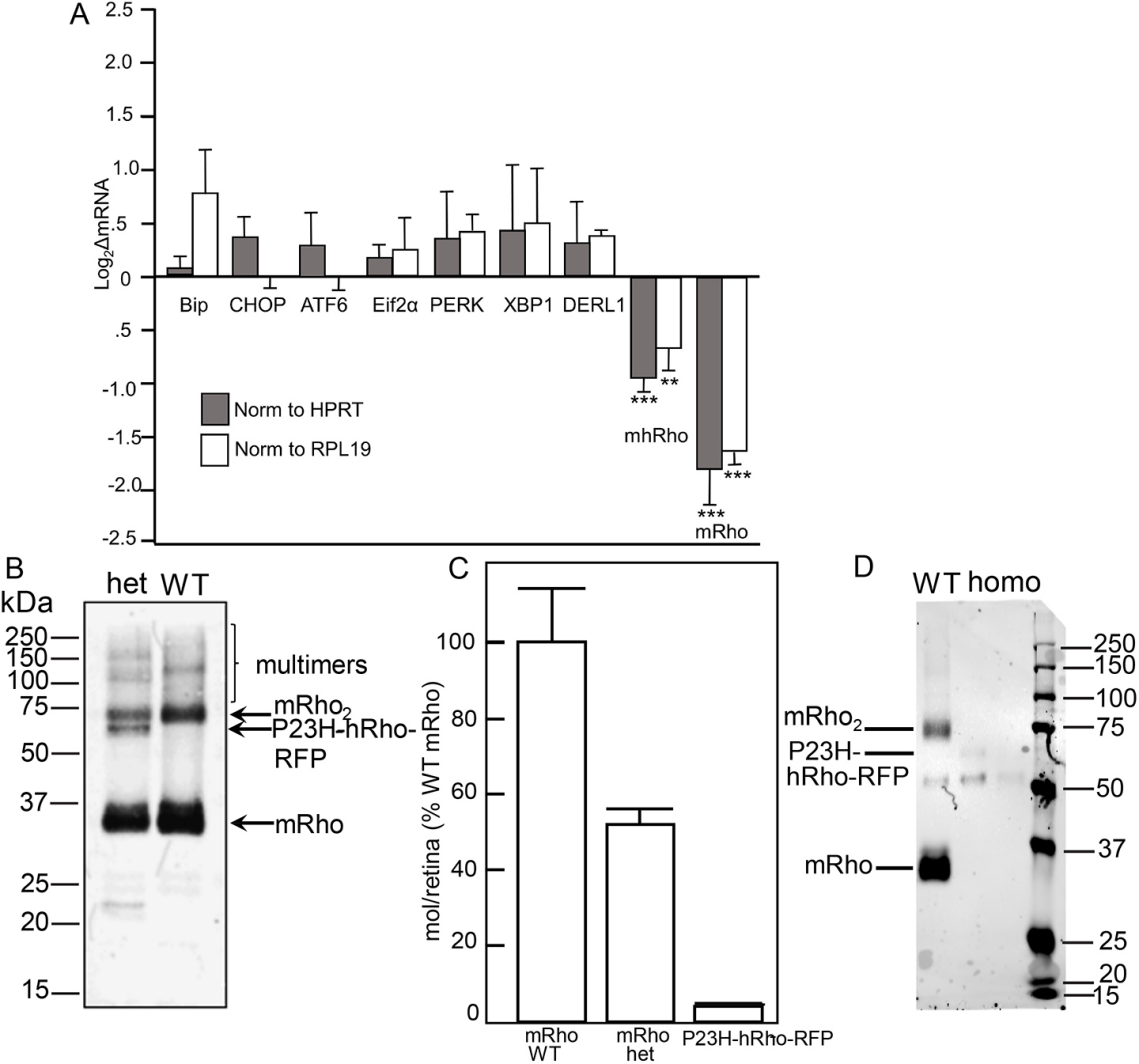
**Alterations of mRNA and protein levels in P23H-RFP/+ het retinas.** (A) mRNA levels of endoplasmic reticulum (ER) stress and unfolded protein response markers are near normal in P23HRFP/+ het retinas at P30. Results of Q-RT-PCR measurements of the indicated messages in RNA extracted from retinas of heterozygotes compared to WT (+/+) (*n*=3 for both), normalized to *Hprt* (dark gray bars) or *Rpl19* (open bars). Statistical comparisons were made with unpaired two-tailed Student's *t*-tests: mhRho (both human and mouse rhodopsin alleles) versus *Rpl19* (**P=0.0099); mhRho versus *Hprt* (***P=0.000741); mRho versus *Rpl19* (***P=0.000585); mRho (mouse rhodopsin allele) versus *Hprt* (***P=0.000542). (B) Image of immunoblot for various forms of rhodopsin. Whole retinas were collected at 4 weeks of age, and 2% of the total protein was loaded onto each lane. After transfer, protein was detected using anti-1-D4 monoclonal antibody and secondary antibody labeled with infrared dye. Levels are adjusted to allow visualization of all bands. (C) Quantification of protein levels by integration of fluorescence intensity. Two samples each from four mice were analyzed for each genotype (total *n*=8). Means and s.d., normalized to WT mouse rhodopsin levels, are shown. Het, P23H-RFP/+; WT, +/+ controls. (D) 2% of the total retina lysates were loaded for one P28 WT retina (‘WT’) and two P23H-RFP/P23H-RFP homozygotes. The signal in the left-hand lane for P23HhRho-RFP protein was less than 2% that of WT mRho in the WT lane. The signal in the region of the mutant protein in the right-hand lane was too low to distinguish reliably from background.

As ER stress and the unfolded protein response (UPR) have been proposed to be important in neurodegeneration due to misfolded proteins in photoreceptors (Gorbatyuk et al., 2020; Chan et al., 2016; Athanasiou et al., 2013; Lin and Lavail, 2010; Newton and Megaw, 2020), we examined mRNA levels for several markers of these pathways, using two different ‘housekeeping’ genes for normalization, *Rpl19* and *Hprt*. Genes for which we quantified messages included those encoding BiP, CHOP, ATF6, Eif2α, PERK, DRL1 and XBP1. None showed a statistically significant increase relative to both ‘housekeeping’ genes (Fig. 8A). We also quantified levels of mRNA transcribed from the rhodopsin locus, using primer pairs that either amplified both human and mouse alleles (mhRho), or the mouse allele only (mRho). Both showed a decrease in message levels in the retinas of heterozygotes relative to those in the WT. The mouse-specific message would be expected to be reduced by ∼50%, based strictly on copy number; however, the reduction was ∼70%, whereas the total message derived from both alleles was down ∼35%, suggesting a downregulation of both *Rho* mRNAs by 35% relative to WT through either reduced transcription or increased degradation, possibly in response to the presence of aggregated protein.

In contrast, levels of the two proteins encoded by the two alleles were very different as determined by quantitative immunoblotting (Fig. 8B,C). The levels of WT mRho in heterozygotes were approximately half those found in WT, as expected from a single allele. The levels of hRho-P23H-RFP were reduced to less than 5% of the mRho levels found in WT, likely due to proteolytic degradation of misfolded protein, as observed in other P23H models (Price et al., 2011; Chiang et al., 2015).

### P23HhRhoRFP protein was misaccumulated in the IS ER

**Fig. 9.**
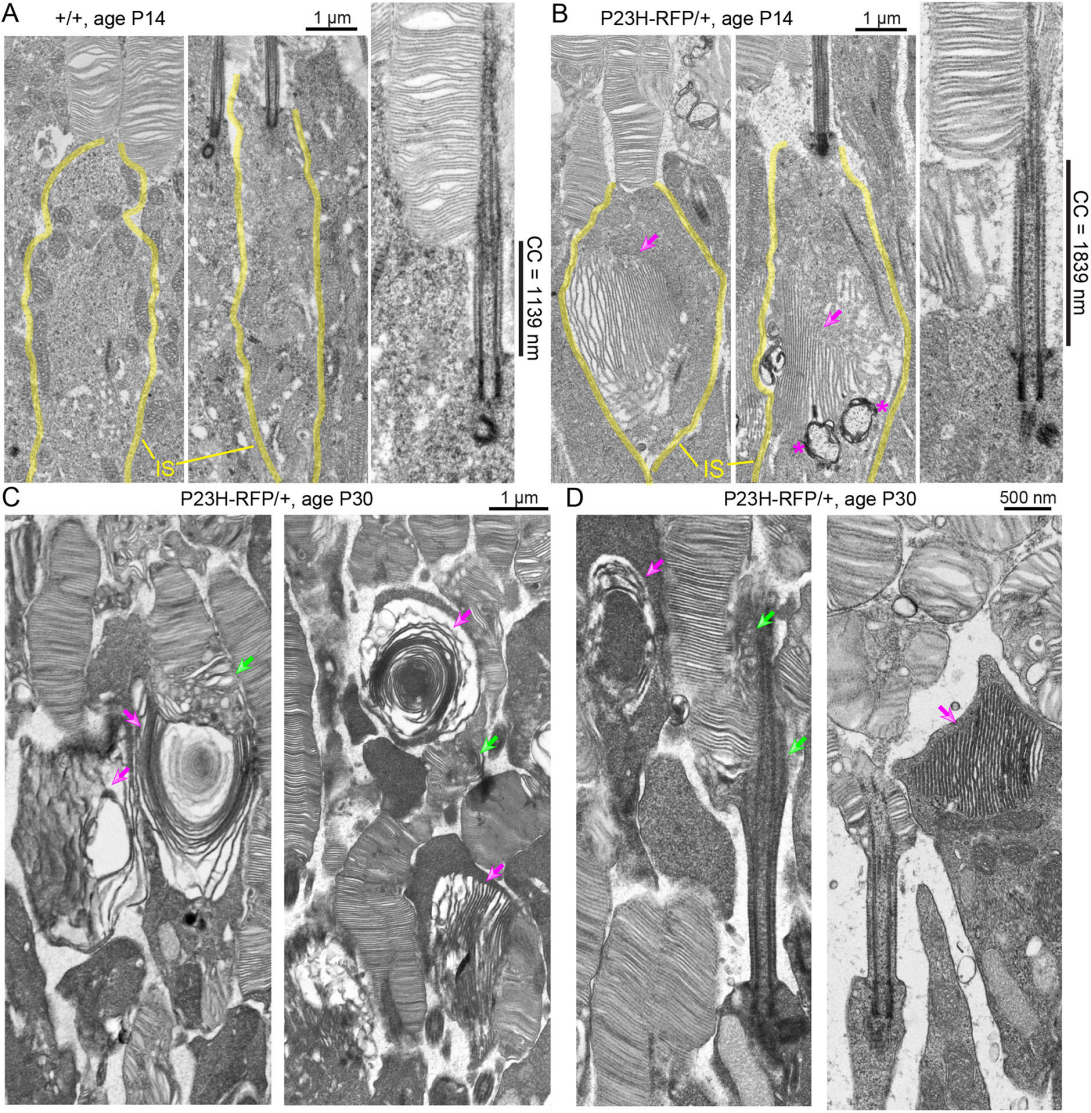
**P23H-Rho-RFP/+ mutant rod photoreceptor neurons have distended ISs filled with ectopic membranes.** (A,B) Transmission electron microscopy (TEM) images of rods at P14 from either (A) +/+ or (B) P23H-RFP/+ het mice. In each example rod, the IS is outlined with yellow lines. The IS maximum width in P14 P23H-RFP/+ rods is significantly greater than that in P14 +/+ rods [het, 2.967±0.508 µm (mean±s.d.) (*n*=14) versus +/+, 1.974±0.481 µm (*n*=19); *P*<0.0001, unpaired two-tailed Student's *t*-test]. Ectopic stacks of IS membranes in the swollen P23H-RFP/+ IS are marked with magenta arrows. Double-membrane autophagy compartments are marked with magenta asterisks in the mutant P23H-RFP/+ IS. In magnified views of example CC from each genotype, the length of the CC – measured by the densely stained CC membrane – is indicated. In aggregate, the length of the CC in P23H-RFP/+ rods is significantly greater than that in +/+ CC [het, 1.548±0.206 µm (*n*=13) versus +/+, 1.27±0.247 µm (*n*=11); *P*=0.0066, unpaired two-tailed Student's *t*-test]. (C) The ectopic IS membranes in P23H/+ rods at P30 appear more dysmorphic compared to those at P14 (magenta arrows). Some OS disc membranes within or adjacent to P23H-RFP/+ rods with IS defects are also disrupted and appear dysmorphic (green arrows). (D) In examples of the CC and basal OS regions of P30 P23H/+ rods, the structure of the CC and BB remain intact despite being adjacent to ectopic IS membranes (magenta arrows); however, there is evidence that basal OS disc morphogenesis is disrupted, possibly due to OS axoneme instability (green arrows).

To understand how the mutant P23HhRhoRFP protein is handled by the P23H-RFP/+ rods, we examined the subcellular structures by transmission electron microscopy (TEM) of ultrathin retina sections following a tannic (Ding et al., 2015) acid-based staining procedure that densely stains internal membranes. At P14, in P23H-RFP/+ heterozygous rods, we observed distinct membranous accumulations in the IS that matched the shape and morphology of the fluorescent RFP^+^ aggregates (Fig. 9A,B). Compared to +/+ rods, in which cytoplasmic membranes were mostly observed in the proximal IS, the membranous accumulations in heterozygous rods were primarily in the form of semi-organized stacks of folded membranes in the distal IS. These membranes often filled the mutant IS cytoplasm, apparently distending the width of the IS itself. Indeed, we measured the average maximum IS width of P14 P23H-RFP/+ rods to be significantly greater than the average maximum IS width of P14 WT rods (Fig. 9, see legend). Despite these large aberrant IS membranes in P23H-RFP/+ rods, the morphologies of the BB, CC and OS discs were relatively normal and indistinguishable between P23H-RFP/+ and WT rods at P14, except for the length of the CC, which was significantly longer in mutant rods (Fig. 9A,B, see legend).

We also used TEM to examine the morphology of the RFP^+^ IS aggregates in P23H-RFP/+ rods at P30, in which the RFP granule fluorescence appeared less organized than in the same rods at P14 (Fig. 4A,B). With TEM, in P30 P23H-RFP/+ rods, we also observed accumulated IS membranes; however, they did indeed appear more dysmorphic than those at P14, with examples of the membranes wrapping around themselves in whorls (Fig. 9C, magenta arrows). As in P14 P23H-RFP/+ retinal sections, the aberrant rod IS membranes at P30 were located among normally formed OS disc stacks, and the CC and BB structures were also morphologically normal at P30 (Fig. 9D). Unlike P14 rods, however, we observed some defects in OS membrane morphology at the base of the OS in some P30 P23H-RFP/+ rods. These defects included vesicular and misshapen discs, and an unbound, splayed OS axoneme (Fig. 9C,D, green arrows). These results indicate that the mutant P23H-Rho-RFP leads to massive alterations in IS membrane structure, accompanied by at least partial disruption of OS morphology.

**Fig. 10.**
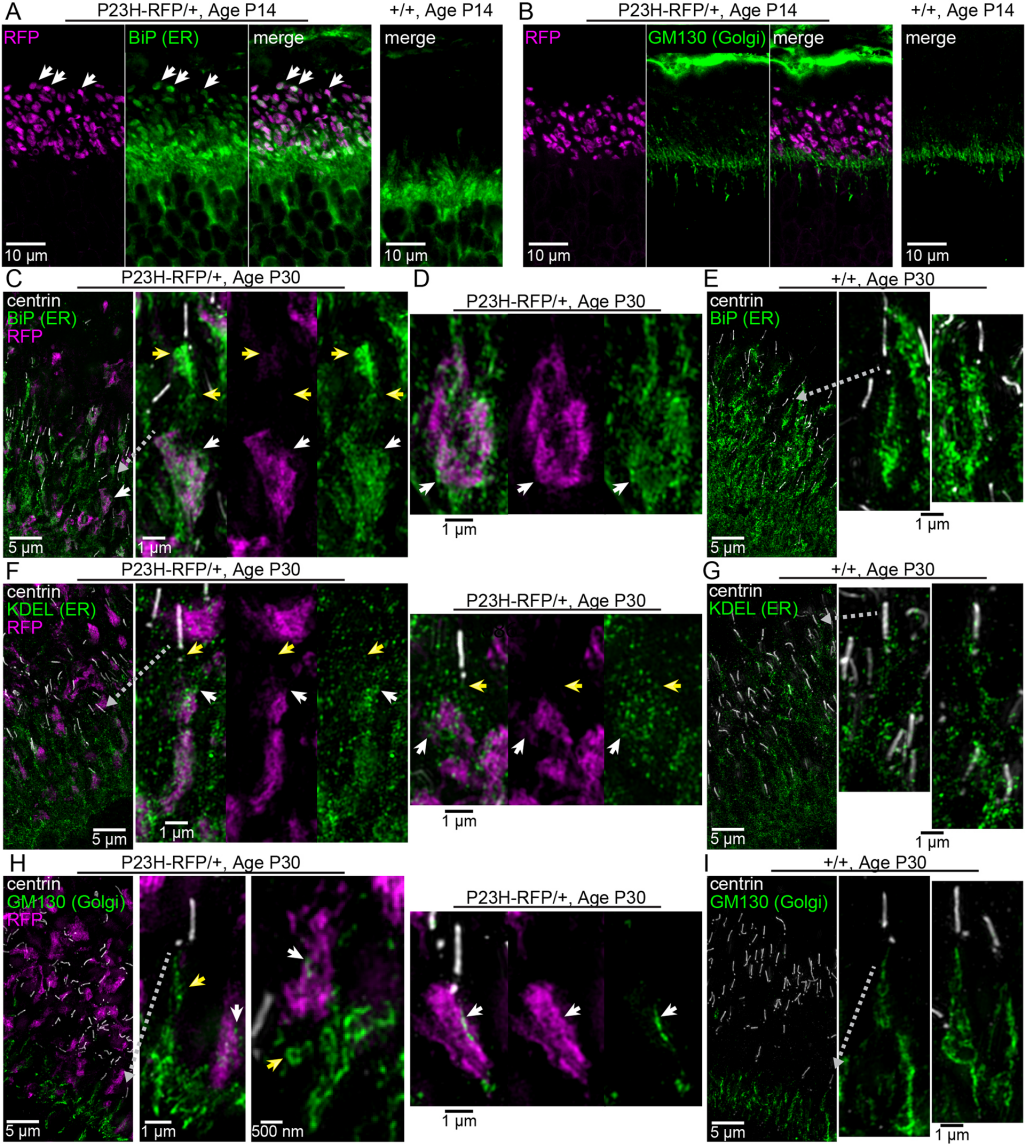
**Mutant P23H-hRho-RFP protein accumulates within ER membranes in P23H-RFP/+ rods.** (A,B) Confocal *z*-projection images of a retinal cryosection from P14 P23H-RFP/+ and +/+ littermate mice, immunolabeled for the ER lumenal marker BiP (green) (A) or the Golgi marker GM130 (green) (B). P23HhRhoRFP fluorescence is magenta. Colocalization of BiP with RFP^+^ aggregates in the P14 P23H-RFP/+ retina (white arrows). GM130^+^ Golgi membranes do not colocalize with RFP^+^ aggregates in the P14 P23H-RFP/+ retina and appear similar in +/+ sections. (C) SIM *z*-projection images of a retina cryosection from P30 P23H-RFP/+ mouse immunolabeled for BiP (green), which labels the ER, and centrin (white), which labels the CC and BB. RFP fluorescence is magenta. In a magnified IS, BiP colocalization with a P23HhRhoRFP aggregate is marked with white arrows. Yellow arrows indicate BiP^+^ ER near the cilium but not colocalized with RFP. (D) Within the same SIM images, BiP^+^ ER tightly surrounds a P23HhRhoRFP aggregate (white arrows). (E) In P30 +/+ SIM control images, BiP immunolabeling marks the IS ER network that leads to the cilium. (F) SIM *z*-projections of P30 P23H-RFP/+ retinal sections immunolabeled for the ER lumen marker KDEL (green), centrin (white) and RFP (magenta). KDEL puncta are more diffuse than BiP, but the localization pattern in the IS is similar; KDEL colocalization with the P23H-RFP aggregates (white arrows) and KDEL^+^ ER near the basal body that is not associated with RFP (yellow arrows) are labeled. (G) In P30 +/+ SIM control images, KDEL immunolabeling (green) labels puncta throughout the inner segment. (H) SIM images of a P23H-RFP/+ retina cryosection at P30 immunolabeled for GM130 (green), along with centrin (white) and RFP (magenta). Overall, the Golgi is proximally localized to the RFP^+^ aggregates at P30, but in some examples the Golgi network reaches the centrin^+^ cilium. On a subcellular scale, the Golgi membranes are generally segregated from RFP aggregates (yellow arrows), but small pieces of Golgi membrane are found colocalized with some RFP aggregates (white arrows). (I) In P30 +/+ SIM control images of retinal cryosections, the Golgi is predominantly dissociated from the cilia; however, examples of +/+ rods with GM130^+^ Golgi membrane networks that reach the BB are shown. Gray dotted line arrows mark a region that is magnified from the same image.

To determine the nature of the membranes in which the RFP fusion protein is located, we used immunofluorescence with antibodies for the ER antigens BiP (also known as HSPA5 and GRP78; an ER lumen chaperone protein) and KDEL (an ER-specific tetrapeptide folding tag), and for the Golgi antigen GM130 (also known as GOLGA2; a Golgi-specific membrane marker). In confocal *z*-projections, we found that BiP ER immunolabeling was colocalized with the RFP^+^ IS aggregates in rod cells from P14 P23H-RFP/+ retinal sections (Fig. 10A). Compared to +/+ BiP labeling, which was largely located in the proximal IS, BiP staining in the mutant P23H-RFP/+ retina was extended into the distal IS and colocalized with the RFP^+^ aggregates. This result demonstrates that the membrane stacks we observed in the IS of mutant rods via TEM are greatly expanded ER membranes. The Golgi network was unaffected in P14 P23H-RFP/+ retinas and was not colocalized with RFP^+^ aggregates (Fig. 10B).

We used SIM super-resolution microscopy to examine the morphology of the ER and Golgi more closely in individual rods in P23H-RFP/+ heterozygous and +/+ retinas at P30. We added centrin immunolabeling to label the CC and BB in these SIM experiments. In P30 P23H-RFP/+ retinas, we again observed BiP colocalization with RFP aggregates in the IS (Fig. 10B,C, white arrows). The BiP^+^ ER lumen surrounded and was intercalated with the mislocalized P23HhRhoRFP protein that appeared aggregated within the ER membranes. We also observed BiP^+^ ER in other regions of the IS, including at the BB (Fig. 10B,C, yellow arrows). In control WT (+/+) P30 retinas, the BiP-positive ER is located throughout the IS in a reticulated morphology that extends to the BB as well (Fig. 10E). We observed similar KDEL^+^ ER localization in P30 P23H/+ rods: both colocalized within RFP^+^ aggregates (Fig. 10F, white arrows) and in the BB region (Fig. 10F, yellow arrows); however, KDEL labeling was more punctate than BiP labeling. In P30 +/+ rods, KDEL was also localized throughout the IS and in the BB region (Fig. 10G). Although there was much evidence of colocalization of RFP and ER marker signal, the ER markers were not uniformly distributed throughout the clumps of RFP signal, and there were large sections of RFP^+^ aggregates without ER marker signal.

We also used SIM to examine the morphology of GM130^+^ Golgi in P30 P23H-RFP/+ rods. As before, we observed the Golgi in the proximal IS of P23H-RFP/+ retinas and segregated from the RFP^+^ aggregates and the centrin^+^ CC/BB. In some mutant heterozygous rod IS, however, we observed smaller GM130^+^ Golgi membranes within the RFP^+^ aggregates (Fig. 10H, white arrows). Also, we found some examples of P30 P23H-RFP/+ rods with more elaborate Golgi that extended into the distal IS and nearby RFP^+^ aggregates (Fig. 10H, yellow arrows). In SIM images of P30 +/+ retina immunolabeled for GM130 and centrin, we found most of the Golgi in the proximal IS and dissociated from the centrin^+^ cilium. Interestingly, however, we did observe some examples of +/+ rods in which the Golgi network was more elaborate and extended up to the BB (Fig. 10I).

**Fig. 11.**
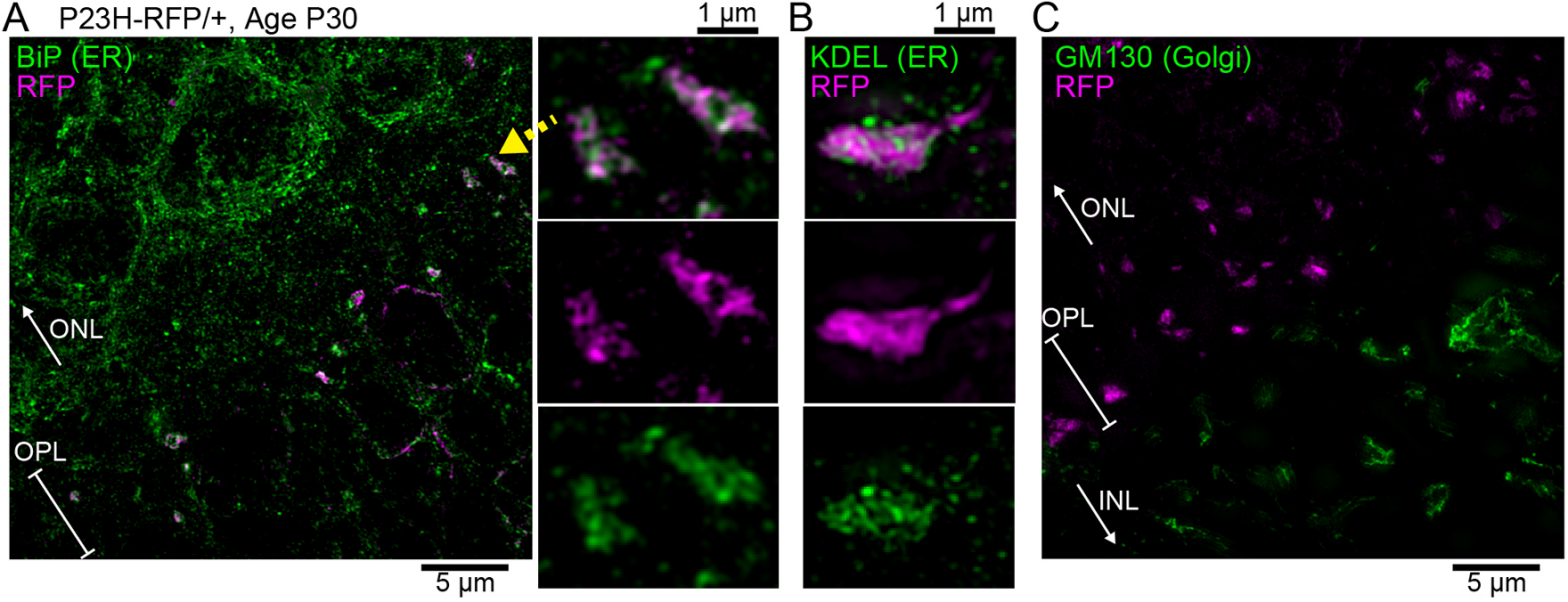
**ER localization of P23HhRh-RFP in the OPL.** (A) SIM *z*-projection image of P23H-RFP/+ retinal cryosection, at P30, immunolabeled for BiP to label the ER (green). P23HRhoRFP protein (magenta) fills the rod photoreceptor synapses in the OPL. BiP^+^ ER labeling is present throughout the OPL and the ONL. In a magnified view of a pair of synapses, P23HhRhoRFP colocalizes with BiP^+^ ER staining. (B) In a SIM magnified view of P30 P23H-RFP/+ retina immunostained for KDEL (green), P23HRhoRFP is colocalized with KDEL^+^ ER staining. (C) Control SIM image: P30 P23H-RFP/+ retina section immunostained for GM130 to label Golgi. No Golgi membranes are evident in the OPL; however, they are present in the INL.

Finally, in our SIM images, we observed both BiP^+^ and KDEL^+^ ER localized with the mislocalized P23HhRhoRFP protein in the region of rod synapses in the OPL of P30 P23H-RFP/+ retinas (Fig. 11A,B). GM130^+^ Golgi was not localized in the OPL of P30 P23H-RFP/+ retinas (Fig. 11C).

## DISCUSSION

The P23HhRhoRFP mouse we introduce here is a potentially useful animal model for adRP that can reveal the subcellular and molecular pathology of the misfolding P23H-Rho mutation on the long-term health of mammalian rod neurons. Tag-RFP-T fluorescence in these mice enables both gross and nanoscopic analysis of P23H-Rho ER accumulation *in vivo*. The WT hRho-GFP/P23HhRhoRFP dual color heterozygote demonstrates the different ways the cell processes these two proteins with similar fusions, but with and without the P23H rhodopsin mutation (Fig. 3). WT-Rho-GFP fusion protein is restricted almost exclusively to the OS in rods of the Rho-GFP heterozygotes (Wensel et al., 2005), whereas the same fusion construct with a P23H mutation is largely confined to the IS and ONL (Price et al., 2011), as observed in the new model reported here, consistent with the notion that the P23H mutation is responsible for the disruption in normal trafficking, likely as a result of misfolding. Furthermore, with super-resolution microscopy, we observed P23HhRhoRFP localization proximal to the CC and BB in P23H-RFP/+ heterozygous rods and completely excluded from the OS (Figs 2 and 3). The exclusive IS mislocalization of the P23H-Rho-RFP protein is unlike P23H-Rho localization in other mouse models, in which there is some detectable OS transport despite other mislocalization and degradation phenotypes (Olsson et al., 1992; Wu et al., 1998; Roof et al., 1994; Sakami et al., 2014).

Although P23H-Rho mislocalized in both P23H-RFP/+ heterozygous and homozygous retinas, rod neurodegeneration homozygotes was more severe. In the heterozygotes, despite a burst of rod cell death around P30, the progression of degeneration in P23H-RFP/+ retinas was partial and relatively slow. By comparison, in the ‘untagged’ P23H-Rho knock-in mouse line, heterozygotes lose 43% of their rod population relative to WT by P63 (Sakami et al., 2011); we did not observe such a severe reduction until P120 (Fig. 5B).

The stability of the P23H-RFP/+ heterozygous rods over time enables long-term studies in these mice and suggests a neuroprotective adaptation despite ER accumulation of P23H-Rho protein. This effect may be related to an ER stress adaptation like the UPR, which was originally characterized based on an increase in *BiP* and C/EBO protein (*Chop*; also known as *Ddit3*) mRNA levels – indicators of activation of the pancreatic endoplasmic reticulum kinase-like endoplasmic reticulum kinase (PERK; also known as EIF2AK3) UPR pathway – in transgenic P23H-Rho rats (Lin et al., 2007). Also in P23H-Rho transgenic rats, overexpression of BiP preserved ERG rod function (Gorbatyuk et al., 2010), and overexpression of the BiP-binding ER chaperone ERdj5 (also known as DNAJC10) preserved photoreceptor survival (Aguila et al., 2020). Although we found no evidence for upregulation of *BiP*, *Chop* or *Perk* at the mRNA level at P30 in P23H-RFP/+ retinas (Fig. 8), we did not measure corresponding protein levels, or mRNA levels, at later ages. We did observe widespread BiP protein in the ER throughout the rod cytoplasm, which colocalized with the ER-accumulated P23H-Rho-RFP protein (Fig. 9).

In contrast, in the untagged P23H-Rho knock-in mouse, the inositol-requiring enzyme 1 (IRE1; also known as ERN1) UPR pathway was shown to be activated concurrent with increased ER-associated protein degradation (ERAD) activity, whereas the PERK pathway was not activated in this model (Chiang et al., 2015). Another study supported a key role for protein degradation by demonstrating that P23H-Rho photoreceptors were preserved by genetically overactivating the proteasome by crossing the untagged P23H-Rho knock-in mouse with mice constitutively overexpressing either the PA28α or PSMD11 proteasomal cap protein (Lobanova et al., 2018). Therefore, the ER membrane expansion we observed in P23H-RFP/+ rods with TEM (Fig. 8) could be a compensatory ER stress mechanism in response to an overload of ERAD and proteasome degradation. Such an adaptation warrants further study. In TEM images of P14 P23H-RFP/+ rods, we also observed double-membrane autophagosome-like structures adjacent to the ER (Fig. 8A), indicating an autophagy component to the P23H-RFP pathology. Similar double-membrane vesiculations were observed in transgenic bovine P23H-Rho *Xenopus* tadpoles that were exposed to light (Bogea et al., 2015), and, more recently, LC3-positive autophagosomes were localized adjacent to P23H-Rho protein in the ISs of these same *Xenopus* rods expressing bovine P23H-Rho (Wen et al., 2019).

We observed a deterioration in the morphology of the ER membranes in P23H-RFP/+ rod IS from P14 to P30, with both RFP fluorescence (Fig. 4) and TEM (Fig. 8). In addition to large IS membrane whorls at P30 (Fig. 8C), the ER membranes were more disorganized than the tight membrane stacks at P14. We also observed ER at the photoreceptor synapse carrying mislocalized P23HhRhoRFP protein in the OPL of P30 P23H-RFP/+ retinas (Fig. 10). Rhodopsin mislocalization to the synapse layer and in the ONL cytoplasm was previously observed in P23H-Rho transgenic mice (Roof et al., 1994). ER may expand throughout the entire cytoplasmic space in rods by P30 as a response to the accumulation of misfolded P23H-Rho protein in the ER.

Although the P23H-Rho mutation leads to protein misfolding in the ER (Saliba et al., 2002; Sung et al., 1991; Kaushal and Khorana, 1994), the accumulation we observe in P23H-RFP mutant rods may be caused by an incompatibility of the mutant P23H-hRho-RFP protein with other components of the rod secretory pathway. Cytoplasmic dynein-1 is the only molecular motor in the rod IS that moves Rho and other cargoes to the BB, presumably via retrograde transport along cytoplasmic microtubules (reviewed in Dahl and Baehr, 2021). In mouse rods, the dynein-1 light chain subunit TCTEX1 and the dynein heavy chain subunit DYNC1H1 are each localized throughout the IS cytoplasm (Dahl et al., 2021; Tai et al., 1999). Bovine-derived Rho-containing vesicles translocate along microtubules *in vitro* (Tsujikawa et al., 2007), while deletion of Dync1h1 and the dynein-1 light intermediate chain subunit Dlic1 have been shown to disrupt normal Rho localization, among other retina morphological defects (Kong et al., 2013). In addition, dynein-1 cargoes, like IS Rho, are recruited to dynein-1 through the cofactor complex, dynactin, which has also been shown to be essential for Rho trafficking and proper photoreceptor development in zebrafish retina (Insinna et al., 2010; Tsujikawa et al., 2007). Thus, the effect of RP mutations like P23H-Rho on the dynein-1 transport system in the rod IS is worthy of future investigation.

Despite the expanded ER membranes filled with P23HhRhoRFP protein, the morphology of the CC and the OS disc structure was normal in P23H-RFP/+ rods at P14 (Fig. 8A,B). At P30, the OS discs in some P23H-RFP/+ rods were slightly dysmorphic, but the CC structure appeared structurally intact, albeit longer than in +/+ rods (Fig. 8C,D). The CC elongation in P23H-RFP/+ rods is surprising. An elongation phenotype was also described in knockout mouse models for two CC-localized proteins: male germ cell-associated kinase (Mak) and huntingtin (Karam et al., 2015; Omori et al., 2010). In both knockouts, the elongated CC was accompanied by aberrant OS morphology and Rho mislocalization. One possible cause of the elongated CC in P23H-RFP/+ mutant rods may be an imbalance of Rho expression during an early ciliogenesis stage due to mutant P23H-Rho-RFP expression.

The rate of cell death observed using TUNEL staining also suggests that P23H-RFP/+ rods undergo a neuroprotective response. Unlike in P23H-RFP/P23H-RFP homozygous retinas, in which the TUNEL^+^ rate in the ONL is elevated at P14, the rate of TUNEL^+^ nuclei spikes at P30 in P23H/+ heterozygous retinas and returns to a level not significantly higher than that in +/+ retinas by P90 (Fig. 7C). A similar spike in TUNEL staining was also observed in both P23H-Rho transgenic rats at P18 versus P30 (Kakavand et al., 2020) and in the untagged P23H-Rho knock-in mouse at P19 versus P31 (Comitato et al., 2020).

In conclusion, our RFP fusion knock-in is a unique model for P23H-associated adRP, with phenotypes traceable with fluorescence microscopy and TEM. The mutant rods in heterozygotes demonstrate a modest rate of retinal degeneration. Our model should be useful to test the variety of proof-of-concept adRP therapies that have been developed across all P23H-Rho models in the field. These include genetic suppression and replacement strategies (Mao et al., 2011; Cideciyan et al., 2018), CRISPR/Cas9 mutant allele deletions (Li et al., 2018; Latella et al., 2016; Giannelli et al., 2018), genetic inhibition of the autophagy-activating *Atg5* (Yao et al., 2018), caspase pathway inhibitors (Comitato et al., 2020, 2016), a small-molecule inhibitor of the photoreceptor-specific transcriptional modulator Nr2e3 (Nakamura et al., 2017), and pharmacological treatments with valproic acid and other histone deacetylase (HDAC) inhibitors (Vent-Schmidt et al., 2017). Future studies that investigate the mechanisms of action for these therapies can be tested in this P23H-RFP mouse for the development of the most efficient and synergistic treatments of P23H-Rho and adRP.

## MATERIALS AND METHODS

### Animals

The P23H-hRho-TagRFP knock-in mice were generated the same way we previously generated P23H-hRho-GFP knock-in mice (Price et al., 2011), by gene targeting in the *Hprt*^−/−^ ES cell line AB2.2 123, which was derived from mouse strain 129SvEv, essentially as described previously (Chan et al., 2004, 2011). The targeting plasmid contained 5′ and 3′ sequences identical to flanking sequences of the mouse *Rho* gene, an intervening sequence corresponding to the human *RHO* gene encoding the P23H mutation found in patients, a C-terminal fusion with the fluorescent protein Tag RFP-T, a final C-terminal peptide corresponding to the C-terminal 9 residues of human rhodopsin (TETSQVAPA, the epitope for the anti-1D4 monoclonal antibody and a putative OS targeting signal), a STOP codon, and an endogenous polyadenylation signal, followed by an expression cassette (minigene) for human *HPRT1*. The *HPRT1* minigene was flanked by loxP sites, so it could be looped out *in vivo* by passing through the germline of GDF-9-iCre mice (Lan et al., 2004) expressing Cre recombinase in oocytes. The plasmid also contained a minigene for herpes simplex virus thymidine kinase (TK) outside the region of homology for negative selection against non-homologous insertion. We introduced the P23H mutation into the targeting vector by site-directed mutagenesis (QuikChange^®^, Stratagene). An ISceI recognition site was engineered into the middle of the first intron in the rhodopsin gene at position 1340 from the start of translation, but it was not used in the experiments described here. The targeting vector was constructed in such a way that there is no lox site between the MOPS promoter and the rhodopsin transcription unit.

The Darwin Transgenic Core Facility, Baylor College of Medicine, electroporated ES cells and injected correctly targeted ES cells (those selected for HPRT^+^TK^−^ genotype) into blastocysts from albino C57BL/6-Tyr^c-Brd^ mice (Zheng et al., 2000). Founder mice carrying the HPRT-P23H-hRho-TagRFP allele were crossed to GDF-9-iCre mice (Lan et al., 2004) to remove the *HPRT1* minigene and screened to ensure germline transmission of the correct targeted sequence without HPRT. P23H-hRho-TagRFP mice were extensively backcrossed to C57BL/6 mice. We validated that the knock-in was successful by sequencing genomic DNA from the knock-in mouse. We verified expression of the P23HhRhoRFP fusion by fluorescence microscopy of retinas and by immunoblotting (Figs 1 and 2).

The P23H-human-rhodopsin-RFP (P23H-RFP) knock-in mice were generated by the Baylor College of Medicine Genetically Engineered Mouse Core, using a strategy similar to the one previously described for our P23H-Rho-GFP knock-in (Price et al., 2011). Homologous recombination in ES cells under positive HPRT selection (HAT medium) was carried out with a plasmid containing 5′ and 3′ sequences identical to flanking sequences of the mouse *Rho* gene, an intervening sequence corresponding to the human *RHO* gene encoding the P23H mutation found in patients, a C-terminal fusion with the fluorescent protein Tag RFP-T, a final C-terminal peptide corresponding to the C-terminal 9 residues of human rhodopsin (TETSQVAPA, the epitope for the anti-1D4 monoclonal antibody and a putative OS targeting signal), a STOP codon, an endogenous polyadenylation signal, followed by an expression cassette (minigene) for human *HPRT1*. The *HPRT1* minigene was flanked by loxP sites, so it could be looped out *in vivo* by passing through the germline of a *Zp3Cre* mouse expressing Cre recombinase in oocytes (de Vries et al., 2000; Lan et al., 2003).

The junction between the mouse and human *RHO* sequences is at the SacI site in the 5′ untranslated region (UTR) between the transcription start sites and the translation start sites. Unlike our P23H-Rho-GFP knock-in described previously (Price et al., 2011), there was no lox site added to the 5′ UTR, although there is a lone loxP site remaining in the 3′ end following loop-out of the *HPRT1* minigene. The sequence of the P23H-hRho-TagRFPr allele was verified by Sanger sequencing. We generated a new hRho-EGFP knock-in mouse line with an additional C-terminal 1D4 epitope sequence tag by a similar approach, using WT human *RHO* sequence and EGFP coding sequence instead of Tag-RFP-T. Generation of the original hRho-EGFP knock-in line was described previously (Chan et al., 2004).

Both P23H-RFP and hRho-EGFP-1D4 mice were extensively backcrossed to C57BL/6 (>10 generations). WT (+/+) C57BL/6 littermates were used as controls throughout this study. The following genotyping PCR primers were used for the P23HhRhoRFP knock-in allele (5′-GTTCCGGAACTGCATGCTCACCAC-3′) and (5′-GGCGCTGCTCCTGGTGGG-3′), which generate a 975 kb knock-in band and 194 bp WT band.

All animal research in this study was approved by the Institutional Animal Care and Use Committee at Baylor College of Medicine, and was carried out in accordance with the guidelines set forth in the Statement for the Use of Animals in Ophthalmic and Vision Research of the Association for Research in Vision and Ophthalmology (ARVO).

### Immunoblotting

Retinal lysates were made by needle extruding mouse retinas in ice-cold cracking buffer [25 mM Tris-HCl (pH 8), 300 mM sucrose, 15 mM EDTA, 2 mM MgCl_2_+1× protease inhibitor cocktail (GenDepot)], and lysates were cleared with centrifugation. Protein concentration was calculated with the BCA assay (Bio-Rad), and sample application buffer was added to lysates, which were sonicated to reduce sample viscosity. Then, 100 µg of each lysate sample was loaded on 10% (Fig. 1), 12% (Fig. 8B) or 12%-14% (gradient; Fig. 8D) acrylamide gradient gels for SDS-PAGE. Gels were transferred onto nitrocellulose in Tris-glycine-SDS buffer and probed with primary antibodies anti-1D4 (Rho) at 1 µg/ml, anti-RFP (Kerafast, 6a11f) at 1 µg/ml or anti-β-actin (Cell Signaling Technology, 8HD10), diluted 1:1000. Mouse monoclonal anti-1D4 (Molday and MacKenzie, 1983) was purified in-house from hybridoma culture medium. Membranes were secondary labeled with one of the secondary antibodies anti-mouse or anti-rabbit IRDye680 (LI-COR Biosciences), diluted 1:10,000, and were imaged on a LI-COR Odyssey imager. For quantitative western blots in Fig. 8B,C, P28 heterozygous P23H-hRFP-RFP/+ and the age-matched WT C57BL/6J mice were dark adapted for ∼12 h. One mouse retina was collected into 200 µl of 2× Laemmli sample buffer (Bio-Rad, 161-0737) containing additional 5% β-mercaptoethanol, 20 mM DTT and cOmplete™ protease inhibitor cocktail (Sigma-Aldrich, 11873580001), and then sonicated on ice for 30 s under dim red light. The retinal lysates were diluted with sample buffer to a final volume of 400 µl, and 2 µl of retinal lysates (0.5% of the total retina) were loaded onto SDS-PAGE gel under room light. After electrophoretic separation, the gels were transferred to nitrocellulose membranes in Tris-glycine-SDS buffer containing 20% methanol. For calibration, rhodopsin standards from 0.0833% to 0.833% of total retina, estimated to contain 0.5-5 pmol mouse rhodopsin (Lyubarsky et al., 2004). In total, two technical replicates from each of four P28 mice were used for each genotype (P23H-hRFP-RFP/+ and WT) (eight lanes for each genotype). For homozygotes, samples of whole-retinal lysates from two 28-day-old animals each of WT and P23H-hRFP-RFP/P23H-hRFP-RFP genotypes were loaded (2% of one retina in each lane). Mouse and human rhodopsin were detected with anti-1D4 antibody (1 µg/ml), and the loading control antibody was rabbit anti-GAPDH (Cell Signaling Technology, 5174S) diluted 1:1000.

Western blot membranes were secondary labeled with one of the secondary antibodies anti-mouse or anti-rabbit IRDye680 (LI-COR Biosciences), diluted 1:10,000, and were imaged on an Odyssey infrared image system (Li-COR Biosciences, Nebraska). All signals were determined to be well within the linear range of the detector. Using ImageJ (Fiji) and Excel, local background values were subtracted from integrated signals for each band, and the resulting net values normalized by the mean value for WT mRho signals on the same gel. For display (Fig. 8B), the minimum and maximum input values from western blot scan images were adjusted to allow visualization of all bands.

### Retinal immunofluorescence

For cyrosectioning, mouse eyes were enucleated and either (1) the cornea was punctured and immersion fixed in 4% paraformaldehyde (PFA) diluted in 1× PBS for 45 min at room temperature before the cornea and lens were removed in 1× PBS, or (2) the cornea and lens were removed, and 1× PBS and eye cups were directly embedded in optical cutting temperature medium (OCT) in plastic cryomolds and flash frozen on a floating liquid nitrogen platform. Fixed eye cups were cryoprotected in 30% sucrose before mounting in OCT in plastic molds and flash freezing. Cryosections (8-10 µm) were collected on poly-L-lysine-coated glass slides (EMS), and unfixed eye cups sections were immediately fixed with 2% PFA for 2 min. This light fixation method was used for all immunolabeling experiments that included anti-centrin cilia immunolabeling. Superior–inferior positions were marked in eyes to be used for retina thickness measurements prior to enucleation to maintain proper orientation throughout fixation and sectioning.

For immunohistochemistry, sections were blocked with either 2% normal goat serum (NGS) (Fitzgerald), 2% bovine serum albumin (BSA) (Sigma-Aldrich), 2% fish skin gelatin (FSG) (Sigma-Aldrich), 0.2% saponin diluted in 1× PBS, or with SUPER block: 15% NGS, 5% BSA, 5% BSA-c (Aurion), 5% FSG, 0.2% saponin in 1× PBS (sections from Fig. 7C,D, Fig. 9 and Fig. 10). Blocked sections were probed with 0.5-2 µg of primary antibodies in the same blocking buffer overnight at room temperature, protected from light. The following antibodies were used: mouse anti-centrin (20H5) (EMD Millipore, 04-1624); rabbit anti-centrin 2 (Proteintech, 15877-1-AP); rabbit anti-BiP (Abcam, ab21685); mouse anti-KDEL (10C3) (Sigma-Aldrich, 420400); mouse anti-GM130 (35/GM130) (BD, 610822); rabbit anti-cone arrestin (EMD Millipore, AB15282).

Sections were washed in 1× PBS and probed with the following secondary antibodies: F(ab′)2-goat anti-mouse/anti-rabbit IgG Alexa 488 (Thermo Fisher Scientific), F(ab′)2-goat anti-mouse/anti-rabbit IgG Alexa 555 (Thermo Fisher Scientific), or F(ab′)2-goat anti-mouse/anti-rabbit IgG Alexa 647 (Thermo Fisher Scientific), diluted 1:500 in blocking buffer, for 1-1.5 h at room temperature, protected from light. Sections were counterstained with 0.3 µM DAPI for 1 h protected from light. Widefield and confocal sections were mounted with #1.5 coverslips in VECTASHIELD (Vector Laboratories), and SIM sections were mounted in ProLong Glass (Thermo Fisher Scientific).

Widefield imaging was performed on an inverted Nikon Eclipse TE2000 U microscope with mercury lamp excitation, a 10× objective (Nikon, Plan Fluor 10×) and imaging via a Photometrics CoolSnap cf Photometrics digital camera (Roper Scientific), with excitation via a mercury lamp using dichroic mirrors and filters for excitation and emission wavelength selection. Full retina sections were generated by merging overlapping captures in Fiji/ImageJ using the ‘Stitching’ plugin (Preibisch et al., 2009; Schindelin et al., 2012). ONL thickness was measured from these full retina section files in Fiji/ImageJ. The position of the optic nerve was designated as position ‘0’; we then traced the DAPI^+^ ONL along the superior (positive nm positions) and inferior (negative nm positions) retina. At each position to be measured, we drew a rectangular region of interest with edges at the top and bottom of the ONL with a constant perpendicular length of 50 µm at each position and collected ONL thickness measurements.

Confocal imaging was performed on either a Leica TCS-SP5 laser scanning confocal microscope with a 63× oil immersion objective (Leica, HC PL APO CS2 63.0×, 1.40 NA) or a Zeiss LSM710 laser scanning microscope with a 63× oil immersion objective (Zeiss Plan Apo 63.0×, 1.40 NA). On both systems, sequential imaging scans with 405 nm diode, 488 nm argon, 543 nm HeNe and 633 nm HeNe lasers were performed with parameters set to capture subsaturation fluorescence and to avoid cross-talk. *Z*-stacks of 1 µm optical confocal sections were projected in Fiji/ImageJ based on maximum intensity values.

SIM imaging was performed on a DeltaVision OMX Blaze Imaging System (v4) (GE Healthcare) with a PLANPON6 60×/1.42 NA (Olympus) oil immersion objective using oil with a refractive index of 1.520. The system features 488 nm, 568 nm and 647 nm laser lines, and a front illuminated Edge sCMOS (PCO) camera. Images were captured in sequential SIM mode with 15 fringe shift images acquired per optical section per channel. SIM reconstruction was subsequently performed in softWoRx 7 software. *Z*-stacks of 125 nm optical SIM sections were projected in Fiji/ImageJ based on maximum intensity values.

All images were pseudo-colored and processed for clarity in Fiji/ImageJ; minimum and maximum input values were adjusted maintaining a linear slope. Magnified images of rod cilia were digitally straightened with the Straighten tool in Fiji/ImageJ.

### ERG

Mice were dark adapted overnight and anesthetized with 90 mg/kg ketamine+14 mg/kg xylazine. For mydriasis, 0.5% tropicamide and 2.5% phenylephrine hydrochloride were added to both eyes. For analgesia/anesthesia, 0.5% proparacaine hydrochloride was used; and 2.5% methylcellulose was used to maintain conductivity and for corneal hydration. A ground electrode was inserted into the mouse forehead, and wire electrode loops were placed over each eye. We used the UTAS BigShot Visual Electrodiagnostic System (LKC Technologies) for ERG recordings. Mice were placed within a Ganzfeld chamber and responses were recorded with a sampling rate of 2000 Hz using a 60 Hz notch filter. Scotopic recordings were averaged from 30 flashes for the intensity range of −55 dB to −10 dB at 5 dB increments. Photopic recordings were collected after adapting the mouse to a constant background light (30 cd/m^2^) for 7 min. Recordings were averaged from 60 flashes for −10 dB and 0 dB intensities, and 20 flashes for 5 dB and 10 dB intensities. We converted intensity values from dB to log[cd*s/m²] values based on the instrument's calibration data.

ERG wave data were visualized and analyzed using a custom Mathematica code previously described (He et al., 2019). Scotopic a-wave amplitude was baseline to the minimum value of the first 35 ms post-flash. Scotopic b-waves were determined after a low-pass filtering at 55 Hz; amplitudes were the a-wave minimum to the maximum of the filtered data between 30 and 120 ms post-flash. Implicit time was the time in ms between the a-wave and the b-wave values. Photopic b-wave waves were also filtered and were baseline to the maximum between 30 and 120 ms post-flash.

### TEM

The following TEM preparation, based on Ding et al. (2015), enhances staining and contrast of internal cell membranes in rod photoreceptors. Mouse eyes for TEM were cornea punctured in the following ice-cold TEM fixative: 2% PFA, 2% glutaraldehyde (EMS), 2.2 mM CaCl_2_ diluted in 50 mM MOPS buffer (pH 7.4), and were then immersion fixed in the same fixative for either (1) 45 min at room temperature with gentle agitation followed by cornea and lens removal and another 1.5 h on ice, or (2) 4°C overnight before cornea and lens removal. Fixed eye cups were embedded in 4% low melt agarose, and 150 µm vibratome sections were cut along the longitudinal plane. Sections were fixed in 1% tannic acid (EMS), 0.5% saponin (Calbiochem) diluted in 0.1 M HEPES (pH 7.3) for 1 h at room temperature with gentle rocking. After rinsing again, sections were stained with 1% uranyl acetate (EMS) diluted in 0.1 maleate buffer (pH 6) for 1 h at room temperature with gentle rocking.

Sections were then dehydrated in the following ethanol series: 50%, 70%, 90%, 100%, 100%, in half dram glass vials filled with 1 ml dehydrant. For Eponate 12 embedding, sections were additionally dehydrated in 100% acetone 2×, for 15 min. Using the solutions of the PELCO Eponate 12 kit with DMP-30 (Ted Pella), a medium hardness resin mix (without accelerator) was prepared and mixed. After dehydrating, the sections were embedded in stages of Eponate 12 resin mix to acetone for the following incubation times: 50%:50% (5 h), 75%:25% (overnight, 16-20 h), 100% resin mix (8 h), 100% resin mix (overnight, 16-20 h). The DMP-30 accelerator was added to the Eponate 12 resin mix just before mixing or incubating. All embedding steps were incubated on a room temperature roller set to a slow speed.

Resin-embedded sections were then mounted in full resin either between two sheets of ACLAR Film (EMS) or in an inverted BEEM embedding capsule (EMS). Mounted sections were cured at 65°C for at least 48 h. Ultrathin resin sections (70-100 nm) were cut on a Leica UC6 ultramicrotome with a Diatome diamond knife. Ultrathin sections were collected onto cleaned 100 mesh copper grids (EMS). Grids for TEM were post-stained on glue sticks first in 1.2% uranyl acetate (diluted in Milli-Q water) for 4-6 min, then in Sato's Lead for 4-6 min after rinsing in boiled Milli-Q water and drying. Grids were rinsed again and dried before storage. Imaging was performed on either a Hitachi H7500 TEM or JEOL JEM-1400 at magnifications up to 25,000×. TEM images were processed for clarity in Fiji/ImageJ by adjusting the minimum and maximum input values (maintaining a linear slope) for contrast and with the Straighten tool.

### RNA extraction and relative quantification of mRNA

Six pairs of retinas from three P30 WT C57BL/6 mice and three P30 P23H-RFP/+ heterozygous mice were collected and homogenized for RNA extraction. A Direct-zol™ RNA MiniPrep Kit (Genesee Scientific/Zymo Research, R2051) was used for RNA extraction, and 25 μl DNase/RNase-free water from the kit was used for elution. RNA extraction product was diluted 20-fold in DNase/RNase-free water (from Zymo Research Direct-zol™ RNA MiniPrep Kit) before recording the UV absorbance spectra on a Hewlett Packard 8452 Diode Array Spectrophotometer. Only RNA samples with 1.7≤A_260_/A_280_ ratio≤2.0 was used for reverse transcription PCR (RT-PCR) experiments. To examine purity, 5 µl of the RNA extraction product was run on a 1% agarose gel. RNA samples with 28S/18S ratio ≥2 were used for analysis. The total RNA input for each RT-PCR reaction was 500 ng. RT-PCR was performed using a LunaScript RT SuperMix Kit (New England Biolabs, E3010L). Each complementary DNA (cDNA) sample from RT-PCR was diluted 100-fold prior to quantitative PCR (qPCR). In the Amplifyt™ 96-Well PCR Plate (Thomas Scientific, 1148B05), 4 μl of the diluted cDNA was added per qPCR reaction followed by 16 μl of master mix with target gene qPCR primers (see primer nucleotide sequences in Table 1), Luna^®^ Universal qPCR Master Mix (New England Biolabs, M3003L) and diethyl pyrocarbonate (DEPC)-treated water. Per qPCR run, three technical replicates were run per biological replicate. Plates were sealed by LightCycler^®^ 480 Seal Foil (Roche Life Science, 04 729 757 001) then spun down in a mini plate spinner. The qPCR reactions were carried out using a C1000 Touch™ Thermal Cycler (Bio-Rad), and the SYBR fluorescent data were collected using the CFX96 Optical Reaction Module for Real-Time PCR Systems (Bio-Rad). Reactions with primers specific for the genes encoding HPRT1 and RPL19 were carried out in parallel for normalization. Data were extracted from CFX Bio-Rad Manager 3.1 as 2007 Excel files. C_q_ values from three technical replicates (with s.d. ≤0.25) were averaged then converted to relative quantification (RQ) value following the Livak method. Graph design and statistical significance analysis of calculated RQ values were carried out using GraphPad Prism 8.4.3. A 95% confidence interval for an unpaired two-tailed Student's *t*-test was used as the criterion for statistical significance. *P*-values for the unpaired two-tailed Student's *t*-tests are reported.

**Table 1.**
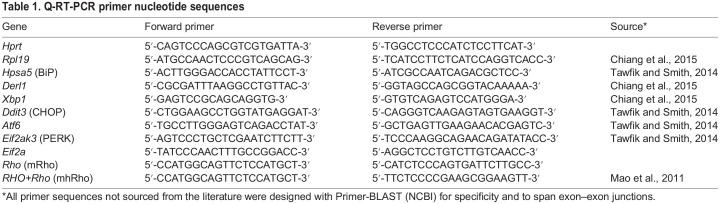
Q-RT-PCR primer nucleotide sequences

### Statistical analysis

Unpaired two-tailed Student's *t*-tests, two-way ANOVA with Šídák multiple comparison tests, and cubic spline curve fittings were performed in GraphPad Prism^®^ software. Each decline in ONL width with age was fitted to a single exponential decay as W(t)=(W(0) – W(∞))exp−(t/τ)+W(∞), where W(t) is the measured width at age, W(∞) is the plateau value to which it declines, W(0) is an initial value, and τ is the time constant. The data were fitted to the equation with W(0), W(∞) and τ as floating fit parameters in a Levenberg–Marquardt least-squares fitting algorithm using Prism.
